# Transcriptome Changes of *Escherichia coli, Enterococcus faecalis*, and *Escherichia coli* O157:H7 Laboratory Strains in Response to Photo-Degraded DOM

**DOI:** 10.3389/fmicb.2018.00882

**Published:** 2018-05-08

**Authors:** Adelumola Oladeinde, Erin Lipp, Chia-Ying Chen, Richard Muirhead, Travis Glenn, Kimberly Cook, Marirosa Molina

**Affiliations:** ^1^National Exposure Research Laboratory, Student Volunteer, U.S. Environmental Protection Agency, Office of Research and Development, Athens, GA, United States; ^2^Department of Environmental Health Science, University of Georgia, Athens, GA, United States; ^3^National Exposure Research Laboratory, National Research Council Associate, U.S. Environmental Protection Agency, Office of Research and Development, Athens, GA, United States; ^4^Invermay Research Centre, AgResearch Ltd, Mosgiel, New Zealand; ^5^Bacterial Epidemiology and Antimicrobial Resistance Research Unit, U.S. National Poultry Research Center, Agricultural Research Service, United States Department of Agriculture, Athens, GA, United States; ^6^National Exposure Research Laboratory, U.S. Environmental Protection Agency, Office of Research and Development, Athens, GA, United States

**Keywords:** fecal indicator bacteria, gene expression, solar irradiated DOM, oxidative stress genes, RNA-sequencing, reactive oxygen species (ROS), hydrogen peroxide

## Abstract

In this study, we investigated gene expression changes in three bacterial strains (*Escherichia coli* C3000, *Escherichia coli* O157:H7 B6914, and *Enterococcus faecalis* ATCC 29212), commonly used as indicators of water quality and as control strains in clinical, food, and water microbiology laboratories. Bacterial transcriptome responses from pure cultures were monitored in microcosms containing water amended with manure-derived dissolved organic matter (DOM), previously exposed to simulated sunlight for 12 h. We used RNA sequencing (RNA-seq) and quantitative real-time reverse transcriptase (qRT-PCR) to compare differentially expressed temporal transcripts between bacteria incubated in microcosms containing sunlight irradiated and non-irradiated DOM, for up to 24 h. In addition, we used whole genome sequencing simultaneously with RNA-seq to identify single nucleotide variants (SNV) acquired in bacterial populations during incubation. These results indicate that *E. coli* and *E. faecalis* have different mechanisms for removal of reactive oxygen species (ROS) produced from irradiated DOM. They are also able to produce micromolar concentrations of H_2_O_2_ from non-irradiated DOM, that should be detrimental to other bacteria present in the environment. Notably, this study provides an assessment of the role of two conjugative plasmids carried by the *E. faecalis* and highlights the differences in the overall survival dynamics of environmentally-relevant bacteria in the presence of naturally-produced ROS.

## Introduction

Survival of bacteria in the environment is dictated by their ability to grow or persist under diverse abiotic and biotic stressors. In aquatic ecosystems, resource availability, sunlight, temperature, pH, and competition have been shown to be important drivers of bacterial population dynamics (Bradford et al., 2013, 2015; Pachepsky et al., 2014). Of these factors, substrate availability, in the form of dissolved organic matter (DOM), is necessary for bacteria to proliferate in surface waters. Further, DOM transformations mediated by sunlight and microbes can have an interacting effect on bacterial survival (Mostofa et al., 2012; Häder et al., 2015).

The colored fraction of DOM (CDOM) is primarily responsible for absorbance of UV light and production of labile nutrients from refractive fractions of DOM, which can be subsequently used for growth (Bushaw and Zepp, 1996; Moran and Zepp, 1997; Häder et al., 2011). These labile nutrients include low molecular weight nitrogen compounds, such as amino acids, that are of great importance to the biogeochemistry of natural waters and are involved in numerous biochemical processes. Another important transient product of absorption of UV/visible light by CDOM is the formation of reactive oxygen species (ROS) from photochemical reactions involving oxygen (Williamson et al., 2017; Wolf et al., 2018). ROS, including hydrogen peroxide (H_2_O_2_), hydroxyl radical (.OH) singlet oxygen (^1^O_2_), and super oxide radicals (${\text{O}}_{2}^{-}$), are photochemically produced reactive intermediates formed during photo-oxidation of CDOM. Further, phototransformation of labile organic compounds, such as thiols present in surface waters can also result in a net increase in ROS steady state (Chu et al., 2016).

Hydrogen peroxide is uncharged and, unlike other ROS, it can easily permeate the bacterial cell surface. It can also persist in natural waters for periods ranging from several hours to days (Kieber et al., 2003; Mostofa and Sakugawa, 2009). Hydrogen peroxide concentration has a diel cycle, with peak concentrations typically noted at noontime during summer months (Clark et al., 2008). At steady state concentrations, H_2_O_2_ concentrations are reported to range from 6 nM in marine waters up to 3.2 μM in rivers and streams (Mostofa et al., 2013). The concentration of photo-produced H_2_O_2_ in surface water is dependent on DOM source/type and concentration, sunlight irradiance, and antioxidants present (Mostofa et al., 2013). Antioxidants, such as glutathione and cysteine are important scavengers of peroxides and are the major component of peroxiredoxins used by prokaryotes and eukaryotes for peroxide signaling (Dubbs and Mongkolsuk, 2007; Parsonage et al., 2008). Subsequently, in extracellular environment we expect the steady state of H_2_O_2_ concentration to be influenced by the concentration of antioxidants or enzymes with antioxidant activity present.

At high extracellular H_2_O_2_ concentrations, microbes can experience toxicity from significant changes in their cell redox homeostasis. Nevertheless, bacteria have developed elegant systems to relieve themselves of H_2_O_2_-induced oxidative stress (Imlay, 2015b). For example, catalase (*kat*) and peroxidase (*ahp*) genes have been demonstrated to be important genes used by *E. coli* under H_2_O_2_ exposure (Imlay, 2015b); and mutants of *dps* and iron-sulfur clusters (*suf*) have been shown to suffer Fenton mediated DNA damage (Djaman et al., 2004; Lim et al., 2007). The majority of these studies have used laboratory grade H_2_O_2_ which may not represent the form of H_2_O_2_ environmentally-associated microbes are exposed to. In addition, the concentrations used in these studies are higher than reported concentrations in natural waters.

The sources of DOM in aquatic ecosystems are diverse and spatially variable. In watersheds dominated by agriculture, livestock farms or lagoon ponds are a major non-point source of DOM via runoff related processes (Bida et al., 2015; Graeber et al., 2015; Heinz et al., 2015). This form of DOM is of fecal origin and has been broken down by the gut microbiome of warm blooded animals, making them bioavailable for use by extra-intestinal microbes. Microorganisms that can readily utilize this source of nutrients for growth should may be at an advantage. Moreover, elevated levels of fecal indicator bacteria, such as *E. coli* and enterococci in streams and rivers have been attributed to these non-point sources of pollution (USEPA, 2017). The survival of indicator bacteria under sunlight exposure have been extensively studied (Rochelle-Newall et al., 2015), however, little is known on the interacting effects of sunlight, DOM, and ROS.

To understand the role of photo-produced ROS on the survival of specific laboratory bacterial strains, we attempted to isolate the effect of H_2_O_2_ using controlled microcosms of natural water spiked with cattle feces-derived DOM exposed to a solar simulator prior to inoculation with bacteria. Following bacterial inoculation, microcosms were incubated in the dark for up to 24 h. We employed high throughput RNA-seq and qRT-PCR to investigate the expression of transcripts required for H_2_O_2_ detoxification and oxidative stress. In addition, we used the RNA-seq data to identify Single Nucleotide Variants (SNVs) acquired following inoculation into water amended with DOM. We provide insights into the different survival mechanisms used by two important fecal indicator strains for water quality monitoring, and a zoonotic pathogen of public health interest.

## Materials and methods

### Cattle fecal extract preparation

Fresh fecal samples were collected from five cows on a commercial farm in northeast Georgia on July 13, 2013. Fecal samples were composited, homogenized, and made into 1:10 fecal slurry in 0.85% KCl and mixed for 1 h in a hand/wrist shaker. The fecal slurry was then centrifuged twice at 4,000 x g for 10 min and the resulting supernatant was saved and named cattle fecal extract (CFE). CFE was sequentially filtered through 1.2, 0.45, and 0.2 μm pore–sized polycarbonate membrane filters. The dissolved organic carbon (DOC) concentration in CFE was determined using a total organic carbon analyzer (TOC-V_CPH_, Shimadzu, Kyoto, Japan) equipped with auto samplers.

CFE was spiked into autoclaved phosphate buffered water (PBW) microcosms to concentrations mimicking nutrient inputs from direct fecal deposition into streams (i.e., final DOC concentration of 32.48 ± 1.52 mg l^−1^). The absence of bacteria and relevant lytic phages in CFE was confirmed by culturing 100 μl of CFE in Brain Heart Infusion (BHI) broth and by performing phage double agar overlay assay (Adams, 1959), respectively. For the overlay assay, bacterial strains used in the present study were used as phage hosts. Thereafter, CFE/DOM-spiked water was divided into two volumes, with one exposed to solar radiation [Irradiated DOM-spiked Water (I-DOMW)] and the other serving as a dark control [Non-irradiated DOM- spiked Water (N-DOMW)].

### Irradiation of DOM spiked water

**Figure 1:**
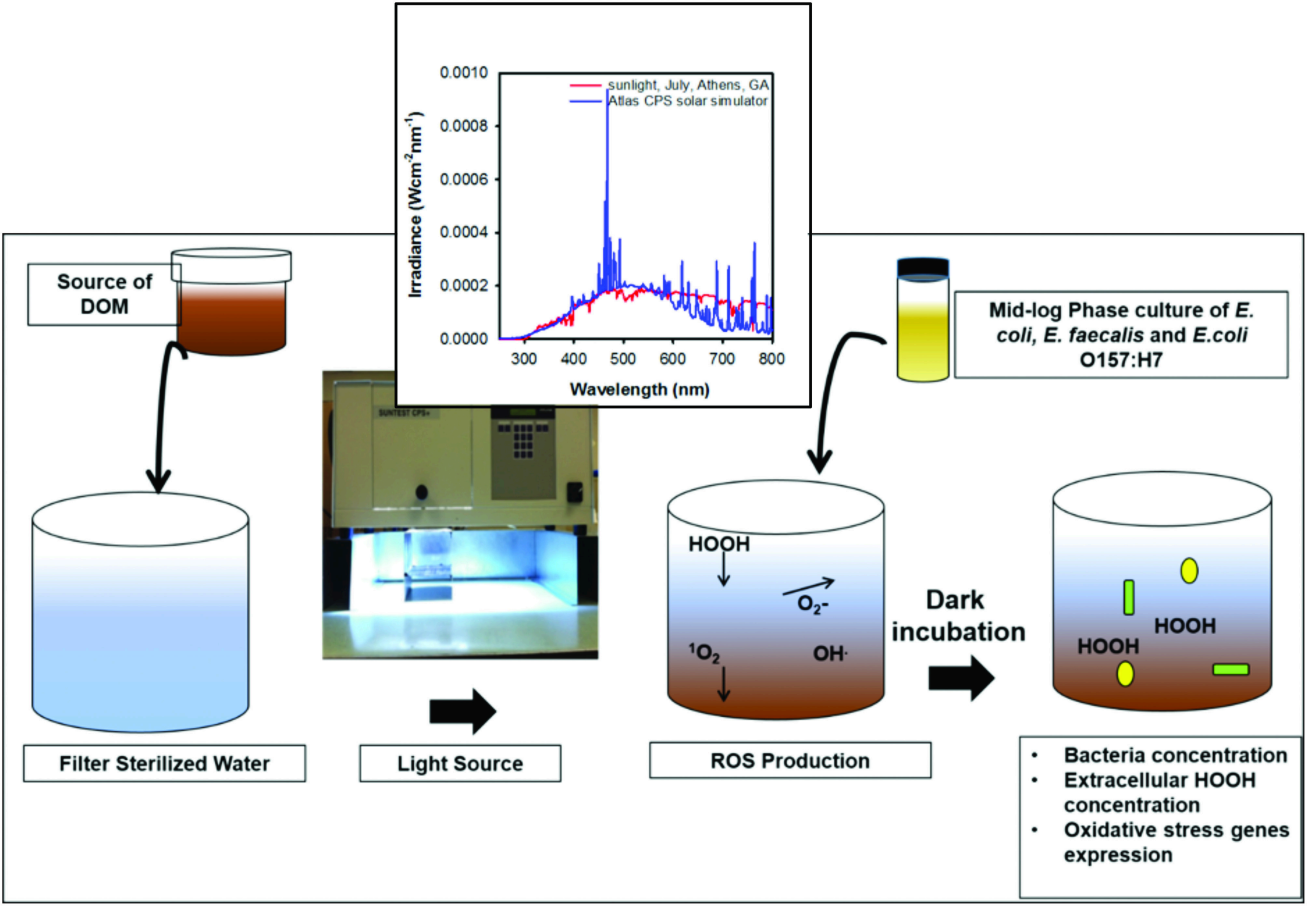
Schematic of experimental design. Inset shows spectra of natural sunlight and light emitted by the Xenon lamp in solar simulator used in this study.

Solar irradiation was performed in an Atlas SunTest CPS/CPS+ solar simulator (Atlas Materials Testing Technology, Chicago, IL) equipped with a 1 kW xenon arc lamp. Irradiance of the simulator in the UV spectral region was similar to mid-summer, midday natural sunlight at 33.95°N, 83.33°W (Athens, GA, USA). Samples were irradiated for 12 h in 25 ml quartz tubes or in a 1 l jacketed Pyrex beaker (Ace glass, Vineland, NJ) before bacteria inoculation (Figure 1).

### Inoculum preparation

Overnight cultures of *E. coli* C3000 (ATCC 15597)—hereafter referred to as *E. coli*; *Enterococcus faecalis* (ATCC 29212)—hereafter referred to as *E. faecalis;* and *Escherichia coli* O157:H7 B6914—hereafter referred to as *E. coli* O157:H7—were harvested, washed and grown for an additional 1.5 h to exponential phase (OD_600_ of 0.1) in BHI broth. The genotypic characteristics of these strains are reported in Table 1. Each culture was centrifuged at 4,000 x g for 5 min and washed twice in phosphate-buffered water (PBW). Thereafter, serial dilutions were made to the desired concentrations. Two inocula concentration were tested in separate experiments: ca. 10^6^ CFU ml^−1^ for high inoculum and ca. 10^3^ CFU ml^−1^ for low inoculum (Supplementary File 1).

**Table 1:** Characteristics of strains used in this study.

**Bacteria (strain)**	**Plasmid Profile (size)^a^**	**Prophages present^b^**	**Unique Single Nucleotide Variants (SNV)**	**Reference^c^**
*E. coli* (C3000)	IncF^d^	8 defective	89	This study
*E. faecalis* (ATCC 29212)	repUS11 (66 kb) rep9 (41 kb)	7 defective	58	Minogue et al., 2014
*E. coli* O157:H7 (B6914)	ND	ND	ND	Uhlich et al., 2017

*ND, not determined*.

a *Determined using the Center for Genomic Epidemiology's PlasmidFinder program*.

b *Determined using PHAST phage search tool*.

c *Published complete or draft genome*.

d *Incomplete plasmid contig*.

### Microbiological analysis

Following irradiation, I-DOMW, N-DOMW, and PBW controls were separately inoculated with mid-logarithmic phase of each bacterium. For low inoculum experiments, 20 ml of each treatment were dispensed into sterile 50 ml centrifuge tubes and incubated in the dark at 25°C in a temperature-controlled incubator shaker (150 rpm) (Innova 4230, New Brunswick Scientific, Edison, NJ). For high inoculum, 50 ml were dispensed in 250 ml Erlenmeyer flasks and covered with sterile aluminum foil before incubation, as mentioned previously. Three replicates derived from one bacterial population were selected at random for analysis, at 0.5, 6, 12, 24, and 48 h. *E. coli, E. faecalis* and *E. coli* O157:H7 populations were quantified by culture methods using modified mTEC agar (EPA method 1603), mEI agar (EPA method 1600), and MUG *E. coli* O157:H7 agar supplemented with 100 μg ml^−1^ ampicillin (Sigma Aldrich), respectively (Figure 1).

### Reactive oxygen species (ROS) measurement

The production and concentration of singlet oxygen (^1^O_2_) and hydroxyl radicals (.OH) were determined separately, as described by Chen and Jafvert (2010). ^1^O_2_ was monitored via the loss of furfuryl alcohol (FFA) and the pseudo-state concentration of ^1^O_2_ was determined. To detect OH, *p*-chlorobenzoic acid (*p*CABA), an OH scavenger was added. *p*CABA was added at a low concentration (2 μM), allowing the pseudo-steady-state concentration of OH to be calculated. Superoxide radicals (${\text{O}}_{2}^{-}$) was measured with chemiluminescence reagent 2-methyl-6-(4-methoxyphenyl)-3,7-dihydroimidazo[l,2-a]pyrazin-3(7H)-one (MCLA) in a FeLume chemiluminescence system (Waterville Analytical, LLC, Waterville, Maine) (Rose et al., 2008).

Extracellular H_2_O_2_ concentrations, before and after bacterial inoculation, were quantified using the copper-DMP (2,9–dimethyl-1, 10-phenanthroline) spectrophotometric method (Kosaka et al., 1998). Samples were filter-sterilized using a 0.22 μm syringe filter to remove bacteria prior to measuring H_2_O_2_. Absorbance readings in I-DOMW and N-DOMW were normalized against N-DOMW controls (no bacteria inoculation). A calibration curve was constructed by plotting the concentration of known ACS-grade H_2_O_2_ (Sigma Aldrich) solution vs. the absorbance at 454 nm of the product formed by reaction of the solutions with copper sulfate and DMP. Two separate calibration curves were used throughout the experiment for quality control and to ensure reproducibility of results. A more complete description of the methods used to detect and quantify ROS is provided in the Supplementary Material.

### RNA isolation

Duplicate microcosms from one high inoculum experiment were selected randomly at 0.5, 6, 12, and 24 h, and ~45 ml was filtered through 0.45 μm pore size isopore membrane (EMD Millipore, Billerica, MA). Filters were folded inwards and saved in a lysing matrix B tube (MP Biomedical, Solon, OH) containing 600 μl RNAlater (Life Technologies, Grand Island, NY). Tubes were kept at −80°C for 2 weeks prior to total RNA extraction. Duplicate filters per time point were removed with sterile forceps and carefully opened to expose the filter surface. Filters were rinsed twice in cold 1X phosphate buffer saline to remove RNAlater, after which 170 μl of 8 mg ml^−1^ lysozyme were dispensed onto the filter surface. Filters containing lysozyme were incubated at 37°C for 5 min prior to extraction. RNA extraction was performed with the FastRNA spin kit for microbes (MP Biomedical, Solon, OH), according to the manufacturer's instructions, except that the bead-beating step was repeated twice at 6.5 m s^−1^ for 60 s. Total RNA was eluted twice in 25 μl DEPC-treated water for a final volume of 50 μl. Total RNA was concentrated using a Vacufuge (Eppendorf, NY, USA), with no heat treatment for 2.5 h.

RNA pellets were rehydrated with 20 μl DEPC-treated water and treated with 8U Turbo DNA-free kit (Life Technologies, Grand Island, NY) to remove genomic DNA contamination. Recovered RNA was quantified with a Nanodrop ND 1000 spectrophotometer (Thermo Fisher Scientific, MA, USA), examined for quality on an Agilent Bioanalyzer, and stored at −80°C until used for RNA-seq (between 2 and 3 months).

### qRT-PCR

Total RNA (100 ng) from each bacterial population was reverse-transcribed with 100 U of high capacity cDNA reverse transcription kit (Life Technologies, Grand Island, NY), per manufacturer's instructions. Primers were synthesized by Integrated DNA Technologies (Coralville, IA) (Table S1). Quantitative Reverse Transcription PCR (qRT-PCR) was performed on an ABI Prism 7500HT Fast Sequence Detection System (Applied Biosystems, Foster City, CA) with 40 amplification cycles, using Fast SYBR Green PCR Master Mix as a signal reporter. Each reaction was composed of 2 μl of cDNA, 1 μM sense and antisense primers for a total volume of 20 μl. qRT-PCR was run in a Fast 96-well microtiter PCR plate using the following amplification conditions: 1 cycle for 20 s at 95°C; and 40 two-step cycles at 95°C for 3 s and 60°C for 30 s. Specificity of primer pairs was verified by melting curve analysis. qPCR efficiency was tested with serial dilutions of cDNA samples, and all ranged between 1.70 and 2.07.

Expression levels of *idnT, cysG, hcaT, gyrB*, and *gapA* (Michán et al., 1999; Kyle et al., 2010; Zhou et al., 2011a) were evaluated as possible reference controls for the normalization of *E. coli* H_2_O_2_ scavenging gene expression (Table S2). The constitutive genes were carefully considered to identify the optimal normalization gene (from the set of candidates) by the geNorm finder algorithm available under the Python library-Eleven (Vandesompele et al., 2002). Data were analyzed using the 2^−Δ*ΔCT*^ method described by Livak and Schmittgen (2001). For each gene, the ratio of expression in I-DOMW, compared to that in N-DOMW, was normalized to the expression of the top two ranked constitutive genes in *E. coli* (*gapA* and *gyrB*). For *E. faecalis*, this ratio was normalized to the expression of *rpoB* and *gyrB* (Riboldi et al., 2014). To assess for reagent and genomic DNA contamination, no-template and no-reverse-transcriptase controls were included.

### mRNA enrichment

Twenty microliters of DNase-treated RNA was pelleted as described earlier and reconstituted in 15 μl (75 ng to 2.5 μg) 1X TE buffer. Bacterial 16S and 23S ribosomal RNA removal was completed using MICROBExpress Bacterial mRNA Enrichment Kit (Life Technologies, Grand Island, NY) according to manufacturer's instruction, but with half of the suggested reaction volumes. The recovered mRNA was quantified using Nanodrop ND 1000 spectrophotometer (Thermo Scientific, Waltham, MA).

### cDNA library preparation

cDNA synthesis was performed on enriched mRNA (~2–160 ng) using the KAPA stranded RNA-seq library preparation kit (Kapa Biosystems, Inc. MA, USA), with a few modifications. Half of the suggested reaction volumes were used throughout. RNA fragmentation was done in a thermocycler at 87.5°C for 6 min. Adapterama I adapters and primers (Glenn et al., 2016) were used for ligation and PCR amplification reactions at a final reaction concentration of 357 and 250 nM, respectively. For PCR amplification, iTru5 forward and iTru7 reverse primers with unique indexes for sample multiplexing were employed (Glenn et al., 2016). The concentrations of amplicons from different samples were quantified using a Qubit dsDNA HS Assay Kit (Thermo Fisher Scientific, MA, USA). The libraries were pooled in equimolar concentrations and sequenced at the Georgia Genomics Facility using an Illumina NextSeq (150 cycles) Mid Output Flow Cell. A 75 bp paired-end sequencing reaction was performed on a NextSeq platform (Illumina, San Diego, CA, USA). cDNA fragments (~339 bp) were obtained for all 24 biosamples. Reads were submitted to NCBI SRA under submission ID SUB1913975 and bioproject PRJNA341849.

### RNA-seq analysis

Unfiltered fastq sequences of each bacterium were aligned to bacterial reference genomes (*E. coli* k-12- NC_000913, *E. coli* O157:H7- NC_002655, and *E. faecalis*- CP008816) using Burrows-Wheeler Aligner (BWA) (Li and Durbin, 2009) with default parameters. Reference genome CP008816 carries two plasmids of sizes 66,548 (CP008815.1) and 41610 bp (CP008814.1). SAM alignment was converted to BAM (samtools view –bS), sorted by coordinates (samtools sort), and PCR duplicates removed using SAMtools (samtools rmdup) (Li et al., 2009). Mapped reads were counted using BEDTools (multiBamCov –bams) (Quinlan and Hall, 2010). Read counts were exported in a tab delimited file for normalization and differential gene expression (DGE) in R (R Developmental Core Team, 2012). DGE analysis was completed with DESeq2 package (Love et al., 2014). Reads with 0 or 1 count were removed before DGE was performed (see Supplementary File 4 for scripts used).

For each RNAseq data set, genes with an absolute fold change ≥ 2 and adjusted *p*.value of <0.01 were used for further analysis in STRINGDB (Szklarczyk et al., 2014). Proteins corresponding to the obtained gene sets were searched against version 10 of the STRING database to display functional protein-association networks. Interactions with a STRING confidence ≥0.4 (medium and high confidence) were considered. A markov cluster algorithim (MCL) (Van Dongen, 2008) of 2 was used for clustering.

### Whole genome sequencing (WGS)

To identify mutations and plasmids in parental *E. coli* and *E. faecalis* strains used in this study, we performed WGS on DNA extracted from pure colonies. Briefly, archived pure cultures were streaked onto Sheep Blood Agar (Remel Inc, San Diego, CA) and incubated overnight at 37°C for 24 h. Following overnight growth, 5–6 single colonies were randomly chosen and resuspended in 200 μl molecular grade DEPC-treated water. Genomic DNA was extracted from resuspended cells with FastDNA Spin Kit (Mp Biomedicals, Solon, Ohio) according to manufacturer's instructions and quantified with a Qubit Fluorometer (ThermoFisher Scientific).

WGS libraries were prepared using MiSeq Nextera XT library preparation kit (Illumina, Inc., San Diego, CA). Sequencing was performed on the Illumina MiSeq platform with 250-bp paired end reads using the MiSeq reagent V2 (500 cycles). All isolates were sequenced for an average coverage of 100X. Sequence reads were assembled de novo into contigs using SPAdes assembler (Bankevich et al., 2012). Assembled contigs were submitted to the Center for Genomic Epidemiology's PlasmidFinder (Carattoli et al., 2014) to determine existing plasmid replicon types. Prophages were identified using PHAST (Zhou et al., 2011b).

### Single nucleotide variants (SNV) identification

SNVs in parental strains and DOMW-treated populations were identified by mapping unfiltered WGS and RNA-seq reads to closed chromosome and plasmid genome of *E. coli and E. faecalis* available in NCBI under accession numbers NC000913 and CP008816, respectively, using BWA. SAM file alignment, sorting and removal of PCR duplicates was done using SAMtools. Genome Analysis ToolKit (McKenna et al., 2010) with a minimum mapping quality of 30 and a minimum base quality of 30 was used for SNV calling.

### Statistical analysis

Bacterial growth rate was derived from the following equation:

$$\begin{array}{l} dN/dt=kN \end{array}$$

where *N* is the concentration of cells, *t* is the time, and *k* is the growth rate constant. Analyses were performed using R (version 3.4.1). A profile analysis was performed to compare bacterial growth rates (μ_6_ vs. μ _0.5_, μ _12_ vs. μ _6_, μ _24_ vs. μ _12_) between treatments, after which a multivariate analysis of variance (MANOVA) tested for significant differences in growth rate between bacteria incubated in I-DOMW and N-DOMW microcosms (Table S3). Although small sample size can affect the power and homogeneity of the variance test, profile analysis still provides more power than univariate tests (Macedo and Waterson, 2016). Graphs were plotted in SigmaPlot (Systat Software, San Jose, CA). Comparisons between treatments were performed using Wilcoxon signed-rank test. A linear regression analysis was used to model the relationship between bacterial concentration (Log CFU ml^−1^) and extracellular H_2_O_2_ concentration (μM).

## Results

### Bacterial growth potential differed in water spiked with DOM

**Figure 2:**
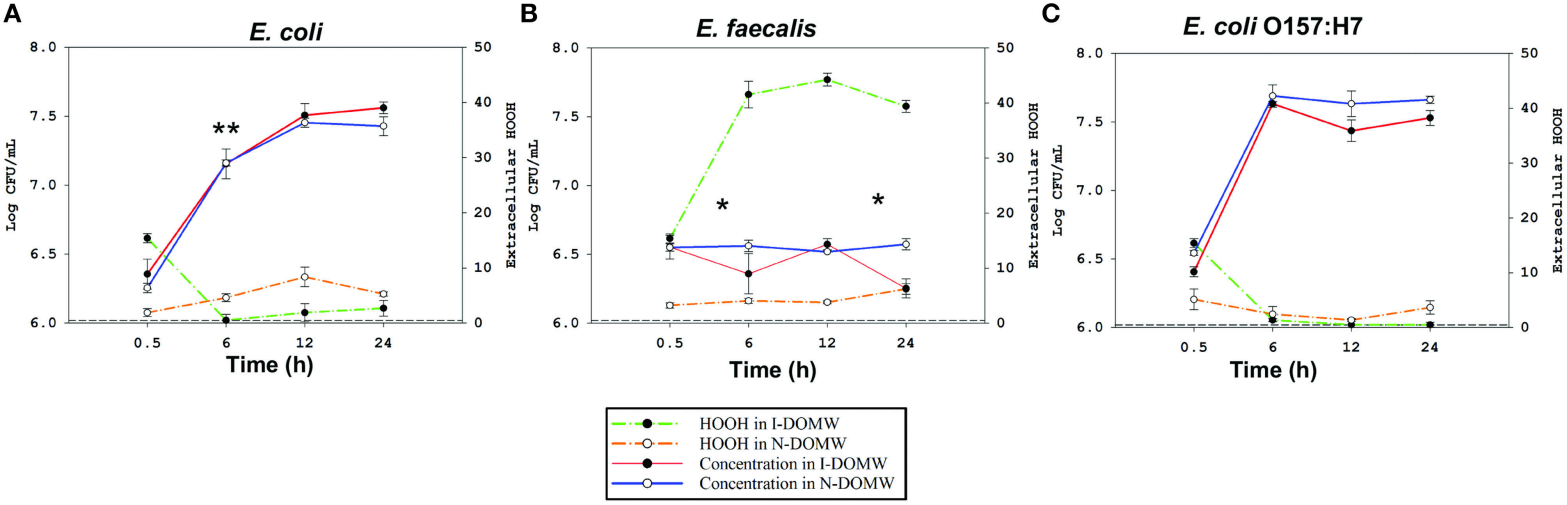
Bacteria concentration and extracellular H_2_O_2_ concentration in the presence of sunlight irradiated cattle fecal extract. A profile analysis followed by MANOVA was performed to test for significant differences in growth rate between bacteria incubated in I-DOMW and N-DOMW microcosms. Dashed lines represent extracellular H_2_O_2_ concentration during dark incubation in I-DOMW (green) and N-DOMW (yellow) in the presence of **(A)** *E. coli* **(B)** *E. faecalis* **(C)** *E. coli* O157:H7. Horizontal short dash lines represent method detection limit for H_2_O_2_ (0.5 μM). Error bars represent standard error for H_2_O_2_ (*n* = 3 replicates per time point) and standard deviation for bacteria concentration (*n* = 2 replicates per time point). ^*^denotes level of significance for the effect of DOM irradiation on bacteria growth rate per time (^*^p < 0.05, ^**^p < 0.01).

To determine the effect of DOM photodegradation on bacterial growth, we inoculated bacteria into DOM-spiked water that was previously irradiated under a sunlight simulator for 12 h. All three strains of bacteria differed in their growth dynamics following inoculation into Irradiated DOM-spiked Water (I-DOMW) and Non-irradiated DOM-spiked Water (N-DOMW). *E. coli* and *E. coli* O157:H7 grew by several orders of magnitude after 12 h of dark incubation in I-DOMW and N-DOMW (Figures 2A,C, Supplementary File 1). At a high inoculum starting concentration (ca. 10^6^ CFU ml^−1^), *E. coli* population had a lower growth rate in I-DOMW than N-DOMW, however, there was no significant difference in maximum concentration achieved when a high or low starting inoculum was used (Table 2). *E. faecalis* population was lower in I-DOMW than N-DOMW after 24 h of dark incubation when inoculated at both low and high starting inocula concentrations (Figure 2B, Table 2).

**Table 2:** Growth rate comparison at low and high inocula concentrations for *E. coli, E. faecalis*, and. *E. coli* O157:H7.

**Bacteria**	**Inocula concentration**	**Treatment**	**Growth rate (hr^−1^)^a^**	**Doubling time (h)**	**Concentration after 24 h (Log CFU ml^−1^)**
*E. coli*	High	I-DOMW	0.14	5.1	7.56
		N-DOMW	0.17	4.1	7.49
	Low	I-DOMW	0.29	2.4	7.49
		N-DOMW	0.24	2.9	7.3
*E. faecalis*	High	I-DOMW	**0.03**	22.6	6.25
		N-DOMW	−0.0024	ND	6.56
	Low	I-DOMW	**0.09**	7.8	5.31
		N-DOMW	0.28	2.5	6.29
*E. coli* O157:H7	High	I-DOMW	0.27	2.6	7.62
		N-DOMW	0.24	2.9	7.72
	Low	I-DOMW	0.26	2.7	7.27
		N-DOMW	0.26	2.7	7.18

*I-DOMW, Irradiated DOM-spiked Water; N-DOMW, Non-irradiated DOM-spiked Water*.

*Boldness denotes significant difference in growth rate between I-DOMW and N-DOMW (p. value < 0.05)*.

*ND, not determined. High and low inocula concentration represents ca. 10^6^ and ca. 10^3^ CFU ml^−1^ of starting bacteria concentration, respectively*.

a *Determined from two separate experiments. Experiments were repeated 8, 3, and 3 X with E. coli, E. faecalis, and E. coli O157:H7, respectively (See Supplementary File 1 for data on experiments not reported in this Table)*.

### Photodegradation of dissolved organic and inorganic compounds

DOM photodegradation resulted in changes in measured concentration of dissolved organic carbon (DOC), ammonium (${\text{NH}}_{4}^{+}$), nitrate (${\text{NO}}_{3}^{+}$), and orthophosphate ${\text{PO}}_{4}^{3-}$. Following 12 h irradiation of DOMW, the concentration of DOC (35.76 ± 11.6 mg l^−1^), [${\text{NH}}_{4}^{+}$] (0.73 ± 0.2 mg l^−1^), and [PO4^3−^] (8.33 ± 1.9 mg l^−1^) decreased to 31.09 ± 5.3, 0.66 ± 0.1, and 8.03 ± 1.8 mg l^−1^, respectively (Table S4). For [${\text{NO}}_{3}^{+}$] (0.44 ± 0.1 mg l^−1^), a 1.2-fold increase was observed after DOMW irradiation (Wilcoxon rank-signed Test, *p*.value = 0.001).

### ROS production and removal in DOM spiked water

The observed significant effect of DOM irradiation on *E. coli* and *E. faecalis* populations (Figures 2A,B), made us question if photo-produced ROS played an inhibitory role. To test this hypothesis, we monitored for the production of singlet oxygen (^1^O_2_), hydroxyl radicals (.OH), superoxide radicals (${\text{O}}_{2}^{-}$), and hydrogen peroxide (H_2_O_2_) following DOM irradiation. Photoproduction of ^1^O_2_, ${\text{O}}_{2}^{-}$, and H_2_O_2_ was observed during 12 h of irradiation of DOMW before bacterial inoculation. No hydroxyl radicals (.OH) were detected. Sunlight irradiation of DOMW produced ^1^O_2_ and ${\text{O}}_{2}^{-}$ at steady states of ca. 2.32^−13^ and 1.3^−9^ M respectively, and H_2_O_2_ at a concentration of 15.38 ± 0.81 μM.

Superoxide radicals are reactive and cannot cross bacterial lipid bilayer, thus its extracellular role in *E. coli* growth rate reduction in this study is unlikely (Imlay, 2008). Regarding ^1^O_2_, we exposed each bacterium to ^1^O_2_ generated from sunlight irradiation of Rose Bengal (See supplementary material for a detailed description of methods used). The steady state of ^1^O_2_ generated (7.38^−13^M) was similar to levels reported in surface waters (Burns et al., 2012) and from DOM irradiation in this study. However, there was no significant difference in concentration between exposed and unexposed populations for the three bacterial strains (Figure S1).

Further, we determined the concentration of extracellular H_2_O_2_ during experiments with high starting concentrations of bacteria. The concentration of photo-produced H_2_O_2_ was negatively correlated with *E. coli* (Adj. *R*^2^ = 0.74; *p*.value = 0.004) and *E. coli* O157:H7 concentrations (Adj. *R*^2^ = 0.93; *p*.value < 0.001) (Figure S2), with extracellular H_2_O_2_ decreasing and bacterial populations increasing in I-DOMW during the first 6 h of dark incubation (Figure 2). On the other hand, extracellular H_2_O_2_ increased significantly in the presence of *E. faecalis*. Extracellular H_2_O_2_ was also detected in μM concentrations with all three types of bacteria during dark incubation in N-DOMW microcosms (Figure 2). Bacterial-produced H_2_O_2_ showed no significant correlation with bacteria concentration (Figure S2), however. There was no significant decline in H_2_O_2_ concentration for I-DOMW controls with no bacteria inoculated (Figure S3).

### Gene expression

Oxidative stress in bacteria is ameliorated by multiple regulatory networks including catalase, peroxidase, cytochrome, and hydrogenase systems. The expression of genes encoding catalase or peroxidase enzymes are regulated based on the ROS level present endogenously and exogenously. For *E. coli*, data showed that steady-state intracellular and extracellular H_2_O_2_ concentration above 200 nM and 2 μM, respectively, are required to induce the OxyR-response (Khademian and Imlay, 2017). To determine highly expressed genes in this study, we performed RNA-seq and qRT-PCR on populations collected from one high inoculum experiment at time 0.5, 6, 12, and 24 h. The number of reads is documented and >90% of reads were mapped to a reference genome (Supplementarty File 2). The average number of reads mapped per sample for *E. coli, E. faecalis* and *E. coli* O157:H7 were 1,806,326 ± 144,075, 1,529,044 ± 124,201, and 1,962,671 ± 446,418, respectively. Mapped reads corresponded to an average coverage of 117 X with a range of 33 to 245 X. The number of genes differentially expressed differed as a function of the type of bacteria and the dark incubation time (Table 3, Supplementarty File 3). Differentially expressed transcripts between I-DOMW and N-DOMW decreased temporally during dark incubation for all strains tested.

**Table 3:** Number of differentially expressed genes (DEG) (*p*.adj < 0.05) between bacteria incubated in I-DOMW and N-DOMW.

**Bacteri**	**Time (h)**	**Upregulated genes in I-DOMW**	**Upregulated genes in N-DOMW**
*E. coli*	0.5	76	88
	6	10	5
	12	3	4
	24	0	0
*E. faecalis*	0.5^#^	22	36
	6	130	143
	12	25	23
	24	2	19
*E. coli O157:H7*	0.5	47	6
	6	21	7
	12	0	0
	24	1	0

*I-DOMW, Irradiated DOM-spiked Water; N-DOMW, Non-irradiated DOM-spiked Water*.

# *p. value reported instead of p. adj due to low sample size (n = 1) at 0.5 h for E. faecalis*.

**Figure 3:**
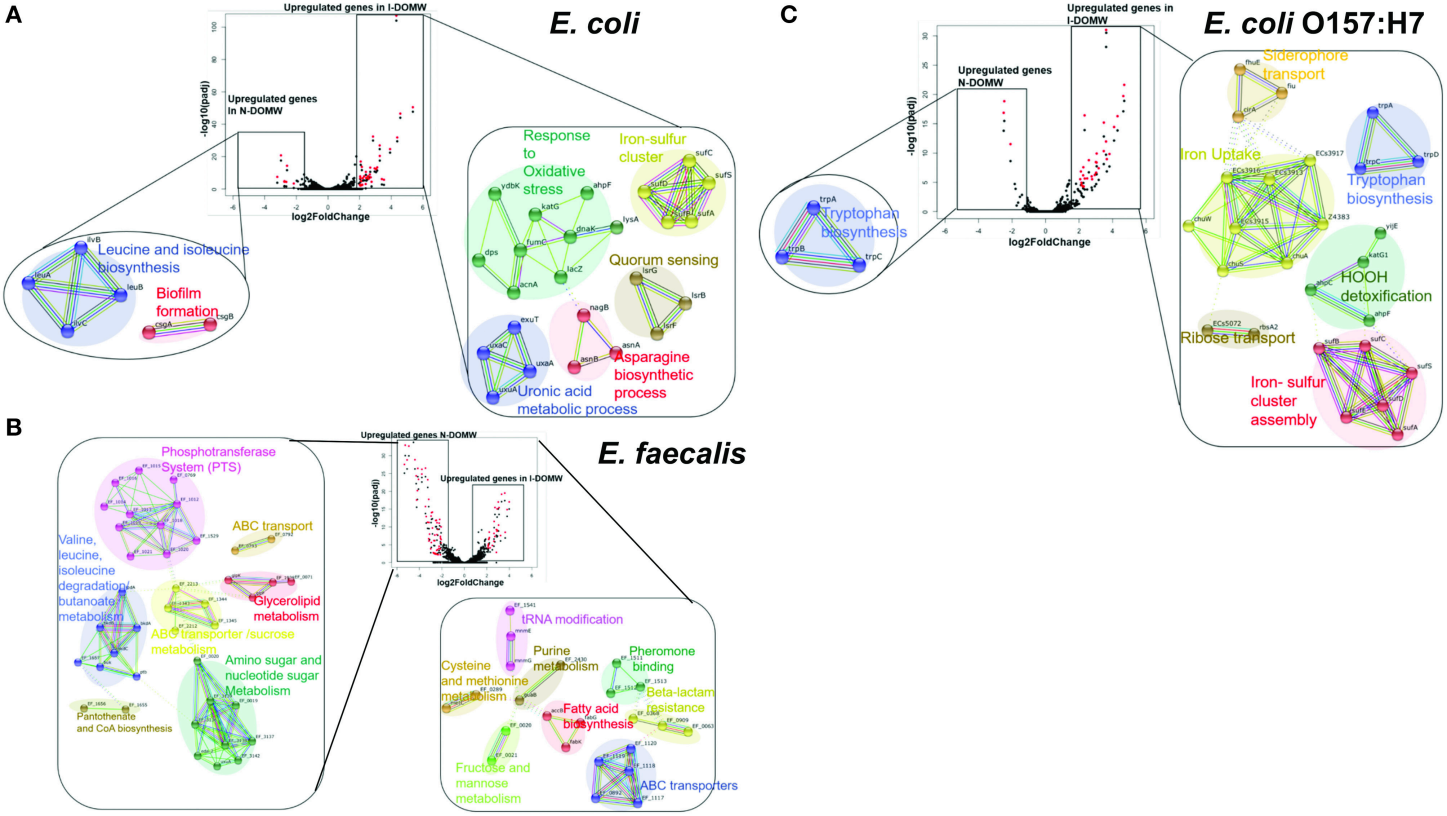
Genetic networks of differentially expressed transcripts during dark incubation as determined by RNAseq. RNA-seq was performed on populations collected at 0.5, 6, 12, and 24 h. Center: Volcano plot showing fold-change of gene expression in I-DOMW compared to N-DOMW. Transcripts with significant increase in expression between the groups (*p*.adj < 0.01; fold- change ≥ 2) are highlighted in red; lower: STRING analysis for significantly altered genes in each case for **(A)** *E. coli* **(B)** *E. faecalis* and **(C)** *E. coli* O157:H7. Solid colored connecting lines depict protein interactions predicted with high confidence (>0.8), while dashed lines indicate protein interactions predicted with moderate confidence (0.4–0.7).

**Figure 4:**
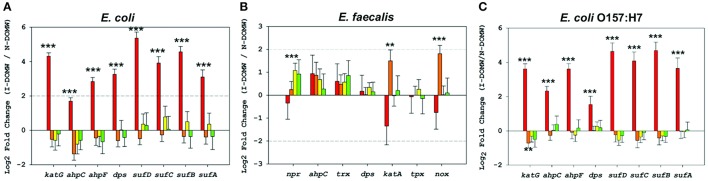
Relative expression of OxyR-type-controlled transcripts by RNA-seq. Fold changes (Log2) in oxidative stress genes for **(A)** *E. coli* **(B)** *E. faecalis* and **(C)** *E. coli* O157:H7 grown in I-DOMW compared to N-DOMW. Error bars represent standard error.

We used STRINGDB to link differentially expressed transcripts to significant genetic interactions (Szklarczyk et al., 2014). The majority of the genes with increased expression for *E. coli* and *E. coli* O157:H7 in I-DOMW were associated with oxidative stress (Figures 3A,C). These genes include *kat* and *ahp* for the detoxification of H_2_O_2_, *sufABCDS* for the repair of iron-sulfur clusters (Fe-S), and *dps* for the sequesteration of irons (Figures 4A,C). In addition, there was increased expression in transcripts involved in iron uptake and siderophore transport in *E. coli* O157: H7. For N-DOMW microcosms, transcripts with increased expression for *E. coli* and *E. coli* O157 were associated with leucine/isoleucine biosynthesis (*ilvBC* and *leuAB*), biofilm formation (*csgAB*) and tryptophan (*trpBCDE*) biosynthesis (Figures 3A,C; Supplementary Figures 4, 6).

**Figure 5:**
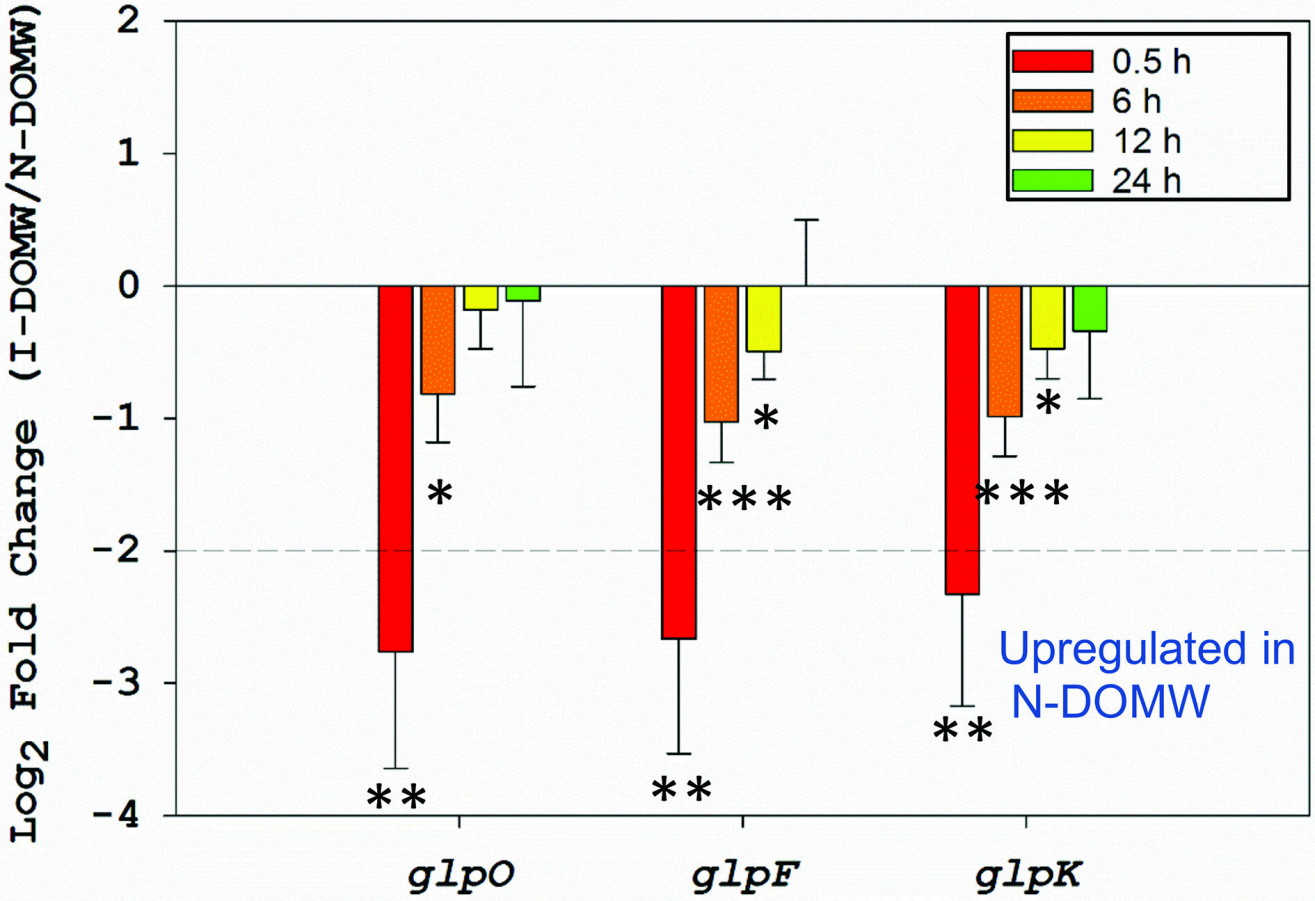
Relative expression of glycerol metabolism genes. Fold-change of genes involved in the aerobic metabolism of glycerol via the *glpK* pathway between I-DOMW and N-DOMW for *E. faecalis* (^*^p.value < 0.05, ^**^ < 0.01, ^***^ < 0.001).

**Figure 6:**
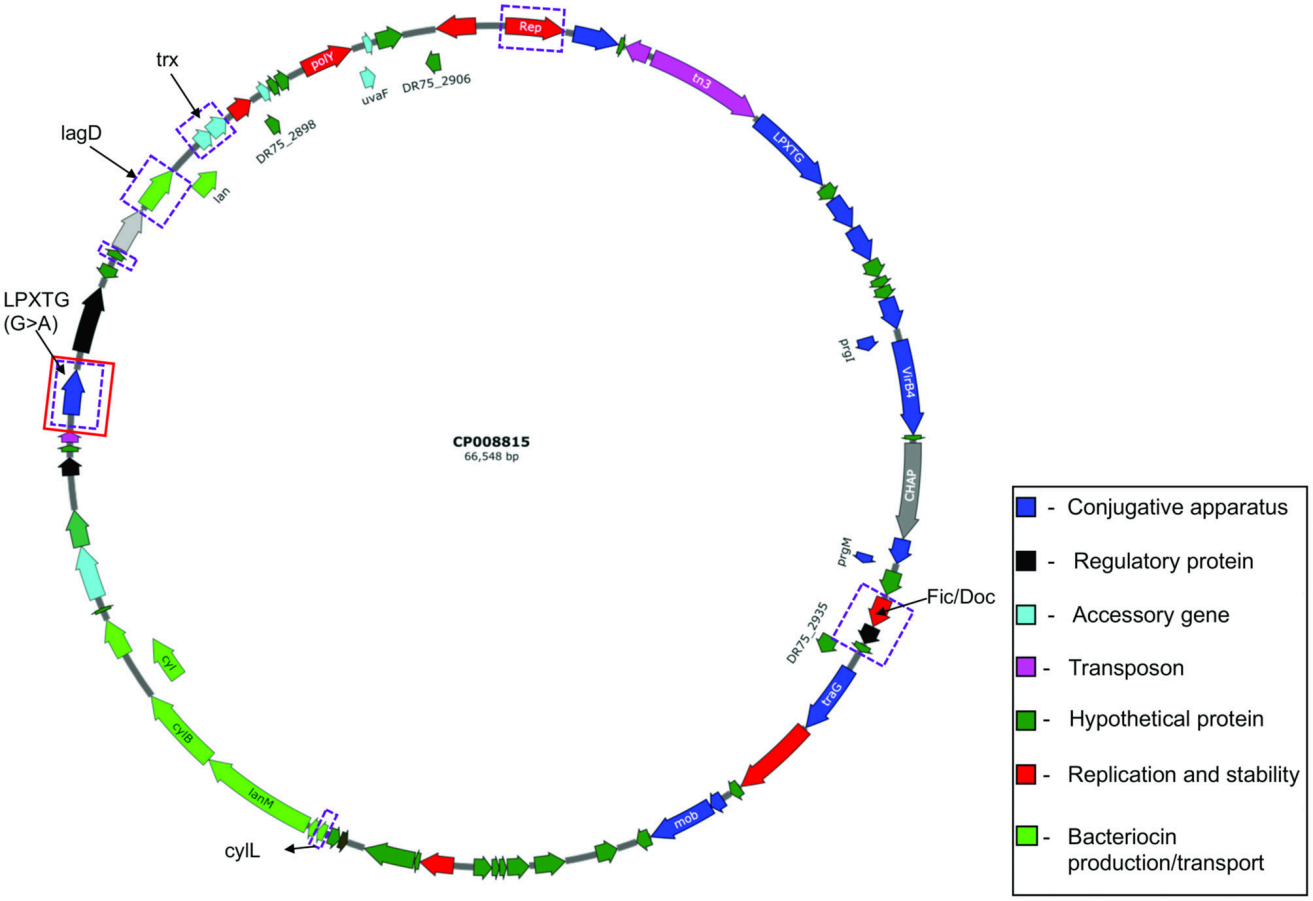
Circular map of *E. faecalis* plasmid pTEF3_66kb (CP008815). Genes differentially expressed are highlighted in purple rectangular boxes and genes with mutations are highlighted in red rectangular boxes.

In comparison, the majority of transcripts with increased expression in I-DOMW, relative to N-DOMW for *E. faecalis*, were associated with ATP-binding cassette (ABC) transporters, fatty acid biosynthesis, pheromone binding and beta-lactam resistance (Figure 3B). Comparatively, glycerol metabolism (*glp*) and sugar transport transcripts were upregulated in N-DOMW compared to IDOM-W (Figures 3B, 5). Twelve plasmid-encoded transcripts were also differentially expressed in *E. faecalis* (*p*.value < 0.05; **Table 5**). Ten of these transcripts were carried on the 66 kb plasmid, and encode genes for replication, recombination, bacteriocin metabolism/transport and thioredoxin (Figure 6). Transcripts associated with replication (*rep*) and bacteriocin production (*cylL*) were the only genes with higher expression in I-DOMW compared to N-DOMW.

Finally, we used qRT-PCR to confirm the relative expression of a few OxyR-like genes (Table 4). *katG* and *ahpCF* genes were the most highly expressed [>2- Fold change (FC)] after 0.5 h for *E. coli* and *E. coli* O157:H7. None of the genes tested for *E. faecalis* were expressed above 2- fold.

**Table 4:** Validation of RNA-seq results with qRT-PCR analysis of selected genes at 0.5 h.

**Bacteria/Gene**	**RNA-seq (Log2 Fold change)**	**qRT-PCR (Log2 Fold change)**
***E. coli***		
***katG***	4.32	5.63
***ahpF***	2.84	3.67
***oxyS***	2.42	4.62
*oxyR*	0.40	−0.029
***E. faecalis***		
*katA*	−1.35	−0.17
*ahpC*	0.94	−0.23
*npr*	−0.34	0.16
*tpx*	−0.066	−0.24
*perR*	−0.51	0.65
*fur*	0.76	−1.55
***E. coli*** **O157:H7**		
***katG***	3.62	5.27
***ahpF***	3.61	6.35
*oxyR*	−0.28	0.20

***Boldness** denotes genes with significant difference in expression between I-DOMW and N-DOMW (p.adj <0.001)*.

### Mutation changes in bacterial populations

To leverage the transcriptome expression with mutations, we identified single nucleotide variants (SNV) on the same data set. In addition, we performed whole genome sequencing (WGS) on parental *E. coli* and *E. faecalis* strains used in this study. Our goal was to differentiate SNVs acquired during dark incubation from those present in parental strains used for inoculation. We did not sequence *E. coli* O157:H7 since transcriptomics results showed that the two *E. coli* strains had similar transcripts differentially expressed and because this strain has been extensively engineered (Fratamico et al., 1997).

Sequenced strains had an average coverage of 113X. *E. coli* and *E. faecalis* differed from the reference genomes used in this study by 89 (NC_000913) and 58 (CP008816) SNVs, respectively, on the chromosome. Additionally, we confirmed that *E. faecalis* carried similar 66 and 41 kb plasmids present in CP008816. These plasmids were identified as repUS11 and rep9, and shared DNA homology with the pTEF3 (accession no: AE016832) and sex pheromone plasmid pAD1 (accession no: L01794) of *Enterococcus*, respectively (Table 1). The number of copies of each plasmid present in a cell was determined using the coverage of assembled chromosomal and plasmid contigs. Based on this calculation (coverage of plasmid contig /coverage of chromosome contig), the pTEF3_66kb and pAD1_41kb plasmids were present at 0.96 ± 0.09 and 1.05 ± 0.19 copies per cell.

*E. coli*_C3000_ carried a multi-replicon IncF plasmid of an unknown size and topology. Two contigs were identified (ca. 14 and 16 kb) from the assembled genome by PlasmidFinder (Carattoli et al., 2014) as carrying the initiation of replication (Rep) protein for IncFIC (FII) and IncFIA plasmids, respectively. DNA sequences from these contigs were subjected to BLAST searches against genomes that have been deposited in GenBank. No BLAST hit matched the complete sequence of the 16 kb plasmid contig, though it shared 99% DNA homology with *E. coli* strain C3026 plasmid (accession no: CP014273). Plasmid C3026 is circular and 213924 base pairs long, suggesting that contigs identified by PlasmidFinder in this study represent only a fragment of a longer IncF plasmid. More importantly, IncF plasmids are usually >100 kb in size (Villa et al., 2010). Due to these factors (unknown size and topology), IncF plasmid will not be discussed further in this manuscript.

**Figure 7:**
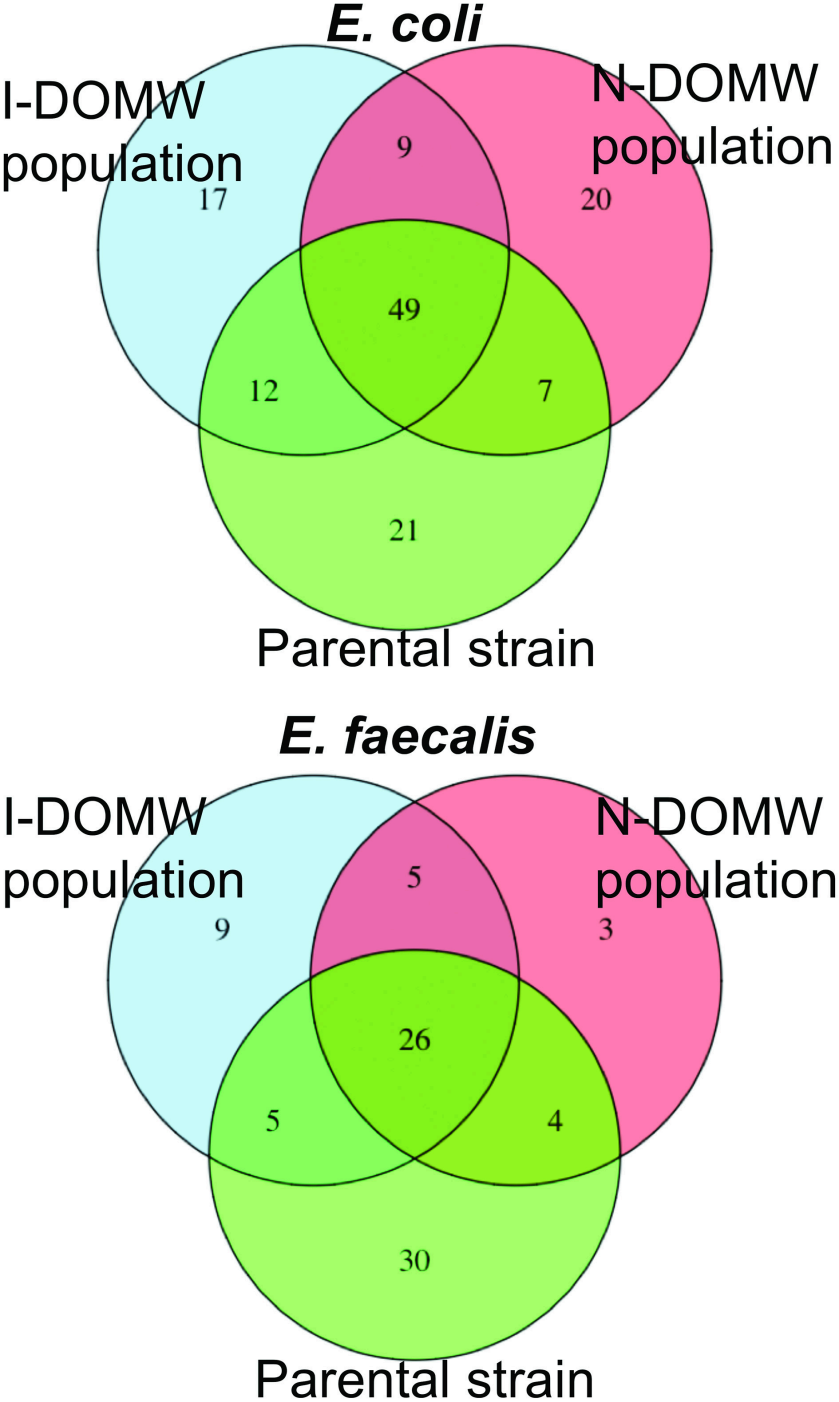
Venn diagram comparing number of Single Nucleotide Variants (SNV) shared between I-DOMW and N-DOMW populations. SNVs for DOMW populations were identified from RNA-seq data and SNVs in parental strains was determined from whole genome sequence.

We identified 17 and 20 high quality SNVs in I-DOMW (*n* = 5) and N-DOMW (*n* = 8) *E. coli* populations, respectively, using RNA-seq data (Figure 7, Table S5A). The mutations in I-DOMW populations were present in genes associated with GTP-binding (*obg, typ*), hydrogen cycling (*hyaB*), and Qin prophage genes (*ydfC*). In N-DOMW populations, these mutations were acquired in genes involved in peptidoglycan metabolism (*dacC, mltA*), oxidoreduction reactions (*Nuo, paoC, nrfD*), and siderophore production (*entC*). For *E. faecalis*, I-DOMW populations (*n* = 6) acquired 9 mutations, whereas N-DOMW populations (*n* = 4) had 3 mutations. I-DOMW populations gained mutations in genes with transferase (*purF, aspB*- locus tag DR75_777 and DR75_1102) and ATP-dependent helicase activity (*rexB*—locus tag DR75_184). *E. faecalis* population in N-DOMW carried a non-synonymous substitution (Asn81Tyr) in *murB* gene involved in peptidoglycan biosynthesis; and a synonymous substitution (Glu269Glu) in a putative LPXTG-motif cell wall anchor domain protein present in pTEF3_66kb plasmid (Table S5B). A significant fraction of the mutations acquired in *E. coli* and *E. faecalis* were absent in populations recovered after 24 h of incubation (Figure S7).

## Discussion

Photodegradation of the DOM of diverse terrigenous sources has been demonstrated to affect the growth and metabolism of bacteria in aquatic ecosystems (Lindell et al., 1995; Scully et al., 2003; Anesio et al., 2005; Lønborg et al., 2013, 2016; Simsek et al., 2013). However, no study to date has provided bacterial transcriptomic or mutational changes occurring after exposure to sunlight irradiated DOM. In this study, we used cattle fecal extract as an agricultural source of DOM to investigate the role of photodegradation on bacterial species used frequently as indicators of fecal contamination; and as control strains in many studies. Based on the maximum concentration and the rapid growth observed in the presence of I-DOMW and N-DOMW, *E. coli* seems to have a greater potential than *E. faecalis* to grow in environments receiving highly bioavailable DOC concentrations (>30 mg l^−1^ C). *E. faecalis* in this study exhibited a steady state or limited growth, suggesting very different requirements than *E. coli*. For instance, Lleó et al. (1998) showed that oligotrophy was the main factor inhibiting divisional capability and activation survival strategies in response to stress caused by low nutrient concentration in *E*. *faecalis, E. faecium*, and *E. hirae*.

Re-growth of *E. coli* in diverse environmental matrices has been reported previously (Sanders et al., 2013; Giannakis et al., 2014, 2015; Harvey et al., 2014; Oladeinde et al., 2014; Oliver et al., 2016). Overnight re-growth has been implicated by high counts of *E. coli* in surface waters in the early morning (Whitman et al., 2004; Desai and Rifai, 2013). Few studies have reported re-growth for *E. faecalis* (Litton et al., 2010; Kim and Wuertz, 2015; Dubinsky et al., 2016), implying that this bacterium might have different survival strategies in the environment. Importantly, the potential for re-growth may be further limited by the inhibitory role of direct sunlight and production of ROS from DOM photodegradation. Under such adverse environmental conditions, *Enterococcus* spp. may favor activation of the viable, but not culturable (VBNC) state, and the loss of culturability (Lleò et al., 2005).

Here, photodegradation of DOM resulted in a decrease in concentration of DOC, [${\text{NH}}_{4}^{+}$], [PO4^3−^], and an increase in [${\text{NO}}_{3}^{+}$]. Although we did not measure all the photo-oxidation products from this reaction, these data suggest that ROS were produced in the presence of oxygen and may have limited bacterial growth efficiency. Following the irradiation of DOM-spiked water for 12 h, ^1^O_2_, ${\text{O}}_{2}^{-}$ and H_2_O_2_ were among the extracellular ROS detected. We discarded the inhibitory role of ^1^O_2_ due to the low concentration detected and short half-life (μs). Additionally, following bacteria exposure to ^1^O_2_ generated from Rose Bengal irradiation, there was no difference in concentration between exposed and unexposed controls.

Maraccini et al. (2016) also reported no correlation between bulk-phase steady state concentrations of ^1^O_2_ and exogenous indirect photo-inactivation rate constants. A few studies have reported on the direct role of ^1^O_2_ in bacterial die-off (Dahl et al., 1989; Sassoubre et al., 2012; Glaeser et al., 2014), but these were done at very high steady state concentrations which may not be representative of environmental relevant doses.

On the other hand, the high reactivity and charge of the radicals limit their potential to cross cell barriers and affect vital cell functions (Kieber et al., 2003). Superoxide radicals cannot cross the lipid bilayers at neutral pH, moreover they are immediately scavenged by superoxide dismutase and reductase enzymes produced by many species of bacteria and cyanobacteria (Imlay, 2008). Hydroxyl radicals is the neutral form of hydroxide ion and it is short-lived. They are formed in a series of Fenton reactions involving iron, H_2_O_2_ and ${\text{O}}_{2}^{-}$ as reactants. Hydroxyl radicals are the most damaging of all ROS, and can cause nucleic acid mutations, lipid peroxidation and amino-acid and protein oxidative damage (Imlay, 2013, 2015a,b). However, the low concentration (< limit of detection) in this study and observed in surface waters (10^−19^–10^−16^ M) (Gligorovski et al., 2015) suggest their extracellular presence is not critical to fecal bacteria survival. For example, Maraccini et al. (2016) reported no correlation between measured exogenous OH^.^ and fecal bacteria inactivation rates in a laboratory-controlled experiment.

The level of H_2_O_2_ produced from DOM photodegradation in this study was in the micromolar range and was stable for more than 24 h (Figure S3). H_2_O_2_ production from DOM photodegradation and microbial processes play a significant role in controlling bacterial survival dynamics in environmental waters (Mostofa et al., 2013; Cory et al., 2016; Zhang et al., 2016). Anesio et al. (2005) showed that the suppression of bacterial carbon production was highly correlated with the concentration of photochemically formed H_2_O_2_ and concluded that extracellular H_2_O_2_ concentrations of about 2 to 3 μM were inhibitory for bacteria. H_2_O_2_ can cross bacterial cell membranes and can persist in surface waters for several hours (Imlay, 2008; Mostofa et al., 2013). In this study, *E. coli* efficiently scavenged photo-produced H_2_O_2_ within 6 h of dark incubation, while there was an accumulation of H_2_O_2_ in the *E. faecalis* treatment. This observed buildup suggests this bacterium differs in its production and detoxification of H_2_O_2_ (Marsico et al., 2015). Moreover, *E. faecalis* produces H_2_O_2_ as a by-product of aerobic glycerol metabolism, which could have contributed to the observed increase in I-DOMW treatment (Bizzini et al., 2010).

### H_2_O_2_ photoproduction and OxyR-response genes in *E. coli* and *E. coli* O157:H7

The OxyR-regulon in *E. coli* is responsible for sensing oxidative stress and positively regulates the induction of several genes including *katG* and *ahpCF* (Zheng et al., 2001; Ravindra Kumar and Imlay, 2013). Mutants lacking either *katG* or *ahp* have been shown to grow poorly or not at all as laboratory cultures (Khademian and Imlay, 2017). Peroxidase genes have the important role of keeping the steady-state concentration of H_2_O_2_ at only 50 nM. This low steady state is necessary since H_2_O_2_ reacts rapidly with vulnerable intracellular enzymes. After bacterial inoculation into I-DOMW, we observed an immediate increase (~0.5 h) in expression of *katG* and *ahpCF* genes (> 2-FC), followed by a concomitant decrease in H_2_O_2_. After 6 h of dark incubation, there was ca. 95% reduction in measured extracellular H_2_O_2_ for *E. coli* and *E. coli* O157:H7.

This does not preclude the presence of hydrogenase encoding genes that can tolerate high oxygen levels and ameliorate H_2_O_2_ induced oxidative stress (Tremblay and Lovley, 2012). For instance, the beta-subunit Ni-Fe hydrogenase protein (HyaB) was identified with a mutation (Val410Ala) in I-DOMW populations after 12 h of dark incubation. This gene was also upregulated (1.85-FC; *p*.value = 0.008) in I-DOMW compared to N-DOMW at 12 h. More studies will be required to correlate this non-synonymous substitution to increased H_2_O_2_ tolerance.

Next, we compared transcripts in *E. faecalis* with a mechanism of action proposed for OxyR by others (Verneuil et al., 2004). Contrary to expectations, peroxidase (*npr, ahpC, tpx*) and thioredoxin (*trx*) genes in *E. faecalis* were not significantly expressed in I-DOMW following inoculation, despite their relevant role in peroxide metabolism (Figure 4B). La Carbona et al. (2007) demonstrated that mutations in these genes can limit survival of *E. faecalis* exposed to exogenously added H_2_O_2_. In contrast, the heme-catalase (*katA*) transcript, which plays only a partial role in protecting *E. faecalis* against the toxic effect of externally-added H_2_O_2_ (Baureder et al., 2012), was significantly upregulated (1.5-FC; *p*.adj < 0.01) in I-DOMW at 6 h (Figure 4B). Further, extracellular H_2_O_2_ concentration increased by 170% after 6 h of dark incubation in I-DOMW, with no significant reduction after 24 h (Figure 2B).

This accumulation of H_2_O_2_ by *Enterococcus* has been demonstrated in several studies (Moy et al., 2004; La Carbona et al., 2007; Bizzini et al., 2010; Baureder et al., 2012). La Carbona et al. (2007) showed that extracellular H_2_O_2_ concentration increased with all carbon sources used for growth, reaching 350 μM in some strains. Further, they reported no difference in growth rate and external H_2_O_2_ (10–20 μM) concentrations between wild type and peroxidase-deleted mutants grown on glucose. A possible explanation could be a requirement for an intracellular peroxide concentration threshold before these genes are activated or highly expressed.

To our knowledge, there is no reported H_2_O_2_ threshold for *E. faecalis*. La Carbona et al. (2007) used >5 mM H_2_O_2_ in their study with *E. faecalis* peroxidase-deletion mutants. Yan et al. (2015) also reported less than a 2-fold change in expression of oxidative stress genes when exposed to 1.5–2 mM H_2_O_2_. These concentrations are ~1000-fold higher than the external levels required for OxyR-response in *E. coli*, and ~100-fold higher than the level produced in this study. It is therefore plausible that in this study, H_2_O_2_ generated inside *E. faecalis* cells was more deleterious than the low concentrations produced exogenously from DOM irradiation. Although a decrease in concentration was observed in I-DOMW treatments at 6 and 24 h (Figure 2B), *E. faecalis* population was the same in I-DOMW and N-DOMW after 48 h (data not shown).

The ability for *E. coli* and *E. coli* O157:H7 to efficiently induce oxidative stress genes in response to ambient levels of ROS may offer them an advantage in the environment. Upon exposure to photo-produced H_2_O_2_, these bacteria are able to remove significant concentrations of H_2_O_2_ and repair DNA lesions within hours, with a potential to grow overnight if necessary substrates are available. Morris et al. (2016), using diel metatranscriptomic data from five published marine studies spanning a variety of open ocean sites, showed that the abundance of transcripts with catalase and peroxidase activity peaked in the late afternoon, coinciding with the highest concentration of H_2_O_2._ Dark production of H_2_O_2_, presumably through the accidental autoxidation of redox enzymes, was observed among all three bacteria in this study. Studies have shown that *E. coli* generates about 10 to 15 μM s^−1^ of endogenous H_2_O_2_ during growth in air-saturated glucose medium (Seaver and Imlay, 2004). Intracellular H_2_O_2_ will accumulate in a closed system by flowing out of the cell rather than into the cytoplasm (Ravindra Kumar and Imlay, 2013). In natural oxic ecosystems (e.g., streams, rivers, and ponds), excreted H_2_O_2_ would not build up, but would be lost to the environment.

This might not be the case for *E. faecalis*; this study shows that while *E. faecalis* can tolerate micromolar levels of exogenous H_2_O_2_, it is unable to reach maximum growth potential. Importantly, such levels of H_2_O_2_ may further limit recovery in the environment; therefore, *E. faecalis* role in H_2_O_2_ concentration fluctuations in surface waters may be restricted to production.

### Glycerol metabolism is associated with extracellular H_2_O_2_ production in *E. faecalis*

**Figure 8:**
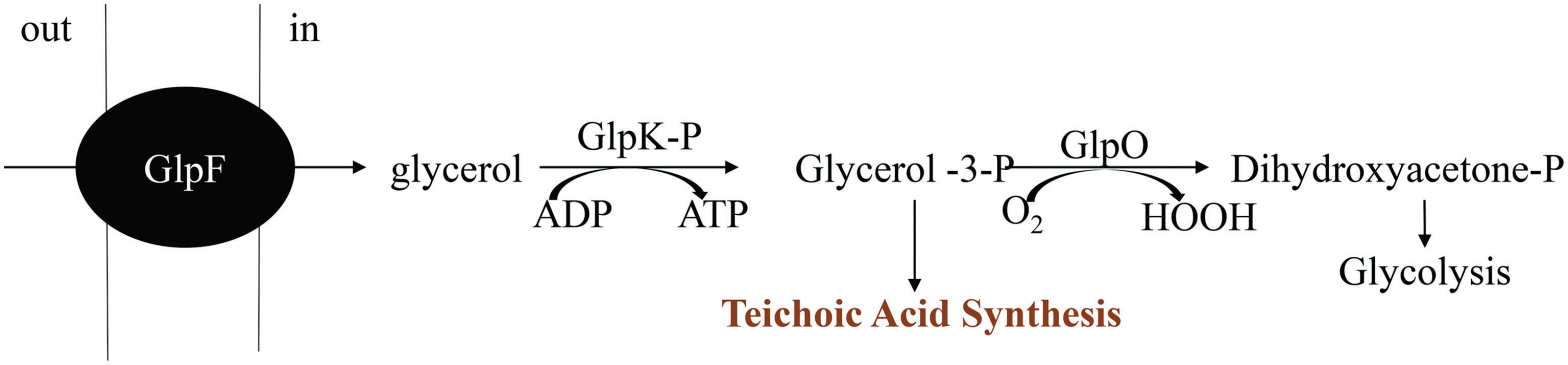
Pathway for aerobic glycerol metabolism. Modified from Ramsey et al. (2014).

The major structural barrier in *E. faecalis* is its peptidoglycan wall that is anchored by teichoic acids (TA) and lipotechoic acids (LTA). TA and LTA are polymers of poly-glycerol phosphate that are joined by phosphodiester linkages (Brown et al., 2013). They provide several functions, including scavenging of cations, phosphate reservoir, cell envelope adhesiveness, and immunogenicity. Glycerol metabolism is important for synthesis of glycerol phosphate in a reaction involving glycerol facilitator protein (*glpF*) and phosphorylated glycerol kinase (*glpK*). The phosphoenolpyruvate (PEP): carbohydrate phosphotransferase system (PTS) is responsible for the phosphorylation of *glpK* (Bizzini et al., 2010; Ramsey et al., 2014). Many of the proteins in the PEP: PTS are involved in transport of sugars including lactose, sorbose, mannose, sucrose, cellobiose, and fructose across the cell membrane. The PTS in *E. faecalis* is also involved in inducer expulsion, inducer exclusion, and catabolite repression (Saier et al., 1996). Hydrogen peroxide production is controlled by glycerol-3-P oxidase (*glpO*) via oxidation of glycerol phosphate to dihydroxyacetone phosphate, an intermediate of the glycolytic pathway (Ramsey et al., 2014). This enzyme uses molecular oxygen as the electron sink, that leads to formation of H_2_O_2_ (Figure 8).

Immediately after inoculation, these glycerolipid transcripts (*glpF, glpO, glpK*, and PEP: PTS enzymes) were significantly upregulated in N-DOMW (compared to I-DOMW) for up to 12 h (Figures 3B, 5), however, extracellular H_2_O_2_ concentration did not significantly change for the duration of our experiment in N-DOMW (Figure 2). This suggests, that *E. faecalis* can tolerate moderate levels of H_2_O_2_ and persist or grow under such conditions. In contrast, H_2_O_2_ increased for 12 h in I-DOMW which indicates the mechanism for extracellular H_2_O_2_ production/detoxification may differ between I-DOMW and N-DOMW.

This increase in H_2_O_2_ in I-DOMW may be due to the activity of *nox*. NADH oxidases (*nox*) use molecular oxygen as an electron acceptor to regenerate NAD+ from NADH. Regeneration of NADH by *nox* allows bacteria to use mixed acid fermentation pathways when grown in the presence of molecular oxygen (Moy et al., 2004). Two categories of NADH oxidases are present in most bacteria—one produces water and the other produces H_2_O_2_. In *E. faecium*, mutation in a Δ*nox* gene eliminated nearly all NADH oxidase activity and reduced H_2_O_2_ production. *Nox* has been shown to be considerably stable in the presence of H_2_O_2_ (Villegas and Gilliland, 1998; Marty-Teysset et al., 2000; Jiang and Bommarius, 2004). Jiang and Bommarius (2004) experimentally determined the inhibition rate constant for H_2_O_2_ was 12 mM for *Lactococcus lactis*, another lactic acid bacteria. In this study, the expression of the *nox* gene was significantly higher (1.8 –FC, *p*.adj < 0.001) in I-DOMW than N-DOMW and was only observed at 6 h (Figure 4B). Further, H_2_O_2_ concentration at 6 h was ca. 42 μM, which is three orders of magnitude lower than the required threshold for *nox* inhibition. Although this may explain the increased H_2_O_2_ production observed in I-DOMW, we cannot rule out the possibility that additional H_2_O_2_-producing genes or metabolic pathways could also be involved (Condon, 1987; Hertzberger et al., 2014).

A negative feedback mechanism in response to the higher external H_2_O_2_ concentration resulting from the I-DOMW treatment may explain the lack of *E. faecalis* growth in the irradiated treatment. A reduction in the expression of *glpK* and *glpF* will restrict the active transport of carbohydrates into the cell and reduce H_2_O_2_ formed via glycerol phosphate oxidation. Although this approach will protect the cell from additional H_2_O_2_ build up, it can limit *E. faecalis's* ability to survive or grow in I-DOMW, which is consistent with the results observed after 24 h of incubation in this treatment.

The sensitivity of *E. faecalis* to light-produced ROS has been reported (Kadir and Nelson, 2014; Nguyen et al., 2015; Maraccini et al., 2016), but its mechanism of survival has not been clearly described. In this study, *E. faecalis* decreased the expression of several proteins involved in glycerol metabolism and transport of carbohydrates, after inoculation into I-DOMW. A consequence of such response was limited growth and accumulation of H_2_O_2_ in the growth medium. Although, *E. faecalis* represents only a species of the genus *Enterococcus*, these results support the opinion that enterococci have a low re-growth potential in environmental waters.

### Conjugative plasmids of *E. faecalis* and associated fitness cost

Plasmids are extra-chromosomal genetic elements coding for a wide range of traits that allow bacteria to adapt to different environmental stressors and can spread horizontally among bacteria by conjugation. However, carriage of multiple plasmids in a host cell has been demonstrated to impose a fitness cost and their long-term stability remains questionable (MacLean and San Millan, 2015). The *E. faecalis* strain used here, carries two low copy conjugative plasmids—pTEF3_66kb and pAD1_41kb; and both encode accessory proteins for class II bacteriocin production (CylL) and a plasmid maintenance system (Par; Fic/Doc, RelB). In addition, pTEF3_66kb carries two thioredoxin peroxidases (*trx*) associated with H_2_O_2_ removal.

Based on the discrepancy in coverage between RNA-seq (mean = 8.3 X) and WGS (mean = 80 X) reads mapping to reference plasmid genomes, these data suggests that plasmid-carrying cells maybe low in I-DOMW and N-DOMW populations. The majority of differentially expressed plasmid-encoded transcripts (>80%) belong to pTEF3_66kb, which implies that there may be a selection pressure for this plasmid (Figure 6). Furthermore, the coverage determined for pTEF3_66kb (10 ± 7.7 X) was significantly higher than the coverage for pAD1_41kb (6.6 ± 4.3X) (Table S6, Wilcoxon rank-signed test; *p*.value = 0.01), supporting the notion that *E. faecalis* selected for it.

Transcripts differentially expressed in pTEF3_66kb were upregulated in N-DOMW compared to I-DOMW which was not expected, since H_2_O_2_ levels were higher in I-DOMW (Table 5). For example, *trx* genes were among the expressed transcripts in N-DOMW after 0.5 h of incubation. A plausible explanation is that pTEF3_66kb *trx* enzyme is more active toward endogenously generated H_2_O_2_ rather than exogenously added H_2_O_2_.

**Table 5:** Differentially expressed transcripts in plasmids carried by *E. faecalis*.

			**0.5 h**	**6 h**	**12 h**	**24 h**
**Locus Tag**	**Plasmid type**	**Gene name/Protein domain**	**Log2 fold change**			
DR75_2888	pTEF3_66kb	LPxTG Gram positive anchor protein	−0.33	−**1.15**	−**0.84**	−**1.02**
DR75_2890	pTEF3_66kb	Hypotethical	−1.08	−**1.69**	**0.86**	0.50
DR75_2893	pTEF3_66kb	Bacteriocin ABC transporter (LagD)	−0.19	−**1.53**	0.35	0.19
DR75_2895	pTEF3_66kb	Thioredoxin	−0.33	−**1.52**	0.32	0.15
DR75_2896	pTEF3_66kb	Thioredoxin	ND	−**2.00**	0.30	0.15
DR75_2908	pTEF3_66kb	Replication protein	0.50	**1.36**	0.11	0.31
DR75_2932	pTEF3_66kb	Fic/Doc family	−0.12	−**0.82**	0.21	−0.36
DR75_2933	pTEF3_66kb	Single strand DNA binding protein (ssb)	−0.01	−**1.11**	−0.01	−0.52
DR75_2934	pTEF3_66kb	putative membrane protein	−0.21	−**1.37**	0.16	0.02
DR75_2953	pTEF3_66kb	cylL	−0.36	**0.71**	**0.75**	0.23
DR75_2989	pAD1_41kb	LagD	−**2.10**	−0.48	0.30	0.09
DR75_2991	pAD1_41kb	Bacteriocin Iic	−1.23	−**2.44**	0.63	0.26

***Boldness** denotes significant differentially expressed genes in I-DOMW relative to N-DOMW (p. value < 0.05). ND, not determined*.

The only mutation identified in *E. faecalis* plasmids was in a putative gene encoding LPxTG cell anchor protein of pTEF3_66kb. This synonymous mutation was observed in N-DOMW populations after 0.5 and 12 h of incubation. Additionally, this gene had significantly higher expression in N-DOMW compared to I-DOMW after 6, 12, and 24 h of incubation (Table 5). This result suggests that codon bias may be in play—a phenomenon in which an organism prefers a different set of codons over others. Codon bias plays an important role in controlling a multitude of cellular processes ranging from fine-tuning gene expression to protein folding (Quax et al., 2015). LPxTG cell anchor proteins have been shown to participate in aggregation substance formation in enterococci and are important for conjugative transfer of virulent plasmids (Hendrickx et al., 2009). Here, this mutation may have contributed to the continuous expression of this gene, which could subsequently increase conjugation rates in N-DOMW populations.

### Extracellular ROS signaling contributes to adaptive response

Environmental cues including non-lethal doses of ROS, antimicrobials, nutrient limitation, and temperature can make bacteria alter their transcriptome rapidly (Kyle et al., 2010; Fernández and Hancock, 2012; Suzuki et al., 2014). Such responses can prolong their survival and enhance resistance to higher doses of the same stressor or other stressors (Dwyer et al., 2014; Djorić and Kristich, 2015). Such adaptive resistance is believed to be transient and usually reverts upon removal of the inducing agent (Fernández and Hancock, 2012).

In this study, multiple genetic networks associated with virulence, quorum sensing, and antibiotic resistance were up-regulated in I-DOMW for *E. coli* O157:H7. These differentially expressed genes included significant increases in genes for outer membrane receptors that facilitate import of iron-chelating siderophores and iron from host organisms which are important for *E. coli* O157:H7 pathogenicity (Figure 3C, Supplementary File 3) (Hagan, 2009). The tryptophan operon is a repressor operon that is turned on or off, based on levels of tryptophan in the environment. Tryptophan is also the primary source of indole production in *E. coli*, an organic compound that plays a role in quorum sensing, biofilm formation and antibiotic resistance (Hu et al., 2010; Kuczynska-Wiśnik et al., 2010). Kuczynska-Wiśnik et al. (2010) showed that the addition of dimethyl sulfoxide (H_2_O_2_ scavenger) partly restored *E. coli* biofilm formation in the presence of antibiotics and decreased indole production.

Transcripts for tryptophan biosynthesis (*trpEDCBA*) increased significantly following inoculation (0.5 h) into I-DOMW and were downregulated within 6 h (Figure 3C and Figure S4). In addition, extracellular H_2_O_2_ concentration in I-DOMW at 0.5 h and 6 h was 15.4 ± 0.81 and 1.34 ± 0.26 (SE) μM, respectively (Figure 2), which was consistent with the gene regulation observed. These results indicate that indole production may be required for *E. coli* O157: H7 survival under increased extracellular peroxide concentration and, consequently, may affect this pathogen's biofilm formation and antibiotic resistance capability.

Curli is a proteinaceous extracellular matrix associated with attachment and biofilm formation, and plays a major role in bacterial pathogenesis (Normark et al., 1992). Moreover, biofilm formation has been associated with oxidative stress, including H_2_O_2_ exposure (Boles and Singh, 2008; Geier et al., 2008; Fink et al., 2012). Extracellular H_2_O_2_ measured at 6 h was higher in N-DOMW than I-DOMW for *E. coli* (4.58 ± 0.71 μM vs. < LOQ) (Figure 2A). Further, the expression of *csgAB* genes encoding curli/amyloid fibers increased significantly in N-DOMW after 6 h of dark incubation (Figure S6). This provides supporting evidence that *E. coli* may increase biofilm formation in response to endogenously-produced H_2_O_2_ (Jang et al., 2016).

The expression of genes, coding for porins, and efflux pumps, are important in adaptive resistant development (Fernández and Hancock, 2012; Motta et al., 2015). They are efficiently regulated to respond to specific cues, thereby changing the resistance of a bacterium based on growth conditions. For example, Suzuki et al. (2014) demonstrated that antibiotic resistance development in *E. coli* could be quantitatively predicted by the expression changes of a small number of genes. Oligopeptide (*opp*) genes encoding sex pheromones (EF0063, EF1513) and peptide transport (EF0909) significantly increased in *E. faecalis* after 6 h of dark incubation in I-DOMW (Figure 3B). These genes have important roles to play in beta-lactamase resistance and quorum sensing in *E. faecalis*, and their induction in response to H_2_O_2_. In the present study, time point 6 h corresponded with a 170% increase in extracellular H_2_O_2_ (Figure 2B), which could have produced the response to oxidative stress observed in the oligopeptide genes. The potential for sublethal levels of ROS to increase bacterial minimum inhibitory concentration (MIC) to antibiotics requires more research. Dwyer et al. (2014) reported that pretreatment of *E. coli* cells with 1 or 5 mM H_2_O_2_ for 15 min did not induce any lethality or growth inhibition, however, it resulted in a transient 1-log protection of cells from antibiotic killing.

Genes associated with quorum sensing (QS) were significantly upregulated in *E. coli* (Figure 3A, Figure S5). QS is a form of regulation of gene expression used by a majority of bacteria in response to fluctuations in cell population density. Quorum sensing bacteria produce and release chemical signal molecules called autoinducers that increase in concentration as a function of cell density. Gram-positive and Gram-negative bacteria use QS communication signals to regulate an array of physiological activities (Rutherford and Bassler, 2012). The level of auto-inducer−2 (AI-2) produced extracellularly varies depending with growth conditions, and its transport is mediated by *lsrACDBFGE* operon. In *E. coli*, expression of *lsrBFG* transcripts significantly increased in I-DOMW after 6 h (Figure S5) which corresponds to the time point with the highest growth rate (Figure 2A). Further, upregulation of QS genes may be influenced by H_2_O_2_ exposure; for instance, Yu et al. (2013) showed that ΔLuxS *Yersinia pestis* mutants (LuxS regulates *lsr*) were more sensitive to killing by H_2_O_2_ than their wildtype.

The survival dynamics shown by pure cultures of bacteria under controlled and sterile conditions in this study could differ in the environment owing to other abiotic factors (e.g., temperature) and biotic factors including predation (“top-down control”) and/or competition (“bottom-up control”) (de Brauwere et al., 2014; Rochelle-Newall et al., 2015). Several studies have suggested that antagonistic agents and indigenous microbiota are important to the survival of indicator bacteria (Feng et al., 2010; Wanjugi and Harwood, 2013; Korajkic et al., 2014). Furthermore, it is a possibility that other free radicals, such as peroxyl radicals and reactive nitrogen species, could have triggered the oxidative stress gene expression exhibited by the bacteria used in this study. Our results suggests that to better describe, understand or model the dynamics of FIB concentration in environmental systems, it is necessary to integrate the effect of abiotic factors, such as the ones discussed herein, with the biotic factors described by others.

## Author contributions

AO was involved in the planning, experimental design, discussion of results, analysis of data, and manuscript writing. MM was involved in the planning, discussion of results, analysis of data, and manuscript editing. RM was involved in the planning, discussion of results, and manuscript editing. EL was involved in the planning, discussion of results, and manuscript editing. C-YC was involved with solar simulation analysis, reactive oxygen species measurement and manuscript editing. TG was involved in preparation of transcriptomics library and bioinformatics analysis. KC contributed to the whole genome sequencing methodology used in this study and also read, revised, and approved submitted manuscript.

### Conflict of interest statement

The authors declare that the research was conducted in the absence of any commercial or financial relationships that could be construed as a potential conflict of interest.
