# Supplementary material for: Transcriptome Changes of Escherichia coli, Enterococcus faecalis, and Escherichia coli O157:H7 Laboratory Strains in Response to Photo-Degraded DOM
Source: Front Microbiol. 2018 May 8;9:882. doi: 10.3389/fmicb.2018.00882 (PMC5953345; doi:10.3389/fmicb.2018.00882)
Supplement: Supplementary file 1 [file Data_Sheet_1.pdf]

Supplementary Material for

**Title: Transcriptome changes of *Escherichia coli, Enterococcus faecalis* and *Escherichia coli* O157:H7 laboratory strains in response to photo-degraded DOM**

Contains:

Supplementary Figures 1-7

Supplementary Table 1-6

Supplementary File 1-4

## SUPPLEMENTARY MATERIAL AND METHODS

***Singlet oxygen measurement***. The production of $^1O_2$ during irradiation of DOMW in the solar simulator was monitored by measuring loss of furfuryl alcohol (FFA). The reaction stoichiometry and steady state concentration calculation has been described in Haag and Hoigne (1986) and Chen and Jafvert (2010). During irradiation, DOMW samples were periodically removed for up to 12 h from light source and analyzed for FFA using high performance liquid chromatography (HPLC).

***Hydroxyl radical measurement.*** *p*-chlorobenzoic acid (*p*CBA) was used as a hydroxyl radical scavenger. The reaction stoichiometry and steady state concentration calculation are described in Haag and Hoigné (1985) and Chen and Jafvert (2010). To measure *p*CBA, filter sterilized DOMW was treated with very low initial *p*CBA concentration (2 µM) prior to irradiation. Residual *pCBA* was measured periodically for up to 12 h by HPLC with UV/Vis detector set at 230 nm.

***Extracellular $H_2O_2$ measurement during dark incubation.*** Extracellular $H_2O_2$ concentration during dark incubation in I-DOMW was quantified using the copper-DMP spectrophotometric method (Kosaka et al., 1998). $H_2O_2$ reduces copper (II) ions to copper (I) ions in the presence of excess 2, 9- diemethyl-1, 10-phenanthroline (DMP). The copper (I) forms a bright yellow cationic complex with DMP at a maximum absorbance of 454 nm. Samples were filter sterilized using 0.22 µm syringe filter to get rid of all bacteria prior to measuring $H_2O_2$. Absorbance reading in I-DOMW was normalized against N-DOMW samples with no bacteria inoculation. A calibration curve was constructed by plotting concentration of known ACS grade $H_2O_2$ (Sigma Aldrich) solution versus the absorbance at 454 nm of the product formed by the reaction of the solutions with copper sulphate and DMP. Two separate calibration curves were used throughout the experiment.

***Rose Bengal photo-sensitization experiment.*** Rose Bengal (RB) was added to sterilized phosphate buffered water (PBW) to a final concentration of 0.05 µM and irradiated under a solar simulator. The production of $^1O_2$ during irradiation was monitored by measuring the loss of furfuryl alcohol (FFA) -- see $^1O_2$ measurement. During irradiation, irradiated RB were periodically removed for 1 h from light source and analyzed for FFA using high performance liquid chromatography (HPLC).

Following irradiation, irradiated RB, non-irradiated RB and PBW controls were separately inoculated with mid-logarithmic phase of each bacterium to a final concentration of $10^2$ - $10^3$ cells $ml^{-1}$. Twenty milliliters of each treatment were dispensed into sterile 50 ml centrifuge tubes and incubated at 25°C in a refrigerated incubator shaker (150 rpm) (Innova 4230, New Brunswick Scientific, Edison, NJ). Duplicate samples per bacterium and treatment were randomly selected for analysis at 0, 0.3, 0.6, 1, 1.3, 1.6, 2, and 3 h.

***Nutrient analysis.*** To determine the concentration of nutrients before and after DOMW irradiation, samples were filtered through 0.2 µm pore size filters and the filtrates were analyzed for nitrate ($NO_3$), ammonium ($NH_4$), orthophosphate ($PO_4^{3}$) and dissolved organic carbon (DOC). [$NH_4^+$] and [$PO_4^{3-}$] were determined by colorimetric methods and [$NO_3^-$] was analyzed as described by Crumpton et al. (1992) using the second-derivative spectroscopy method. DOC was determined using a total organic carbon analyzer (TOC-$V_{CPH}$, Shimadzu, Kyoto, Japan) equipped with auto samplers.

## SUPPLEMENTARY FIGURES

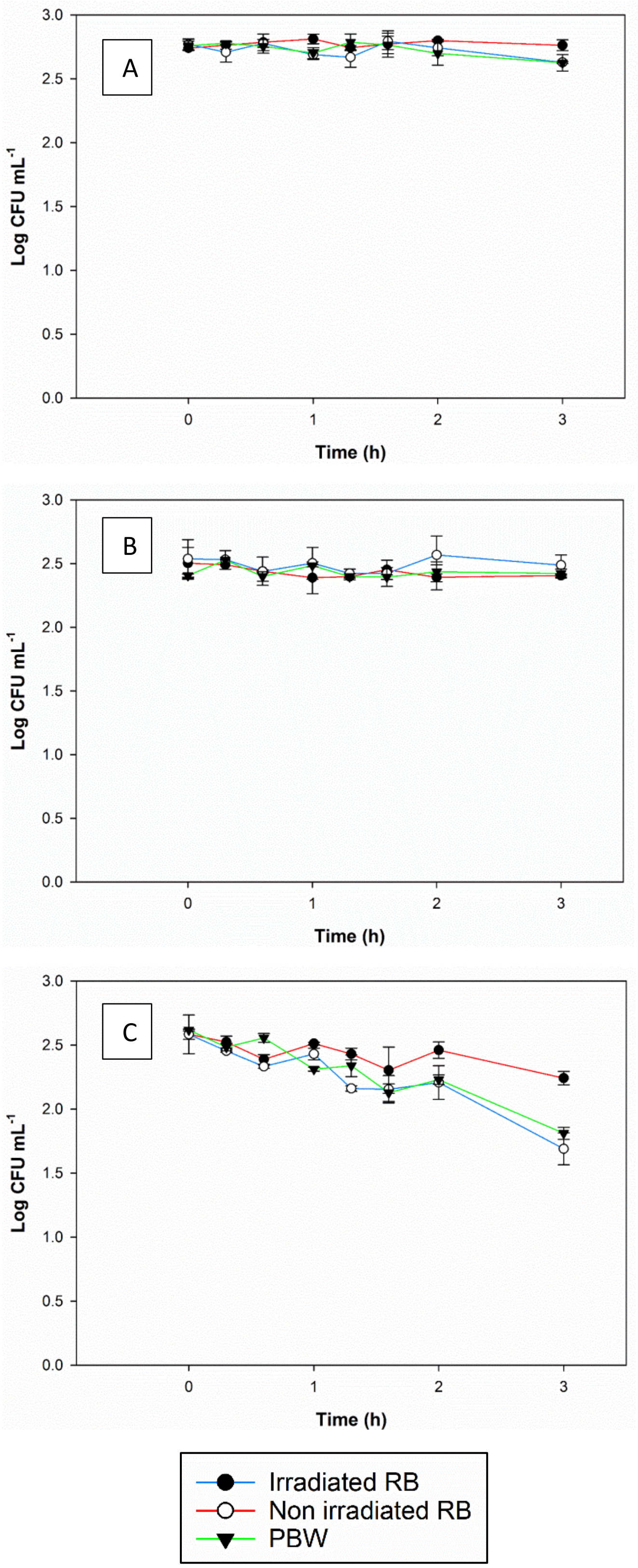

**Figure S1**: **Bacteria concentration after exposure to photo-sensitized Rose Bengal (RB)**. Singlet oxygen was produced at a steady state of $7.38^{-13}$ M after 1 h of sunlight irradiation. Inactivation of (**A**) *E. faecalis* (**B**) *E. coli* (**C**) *E. coli* O157:H7 in photo-sensitized RB. Error bars represent standard deviation.

A

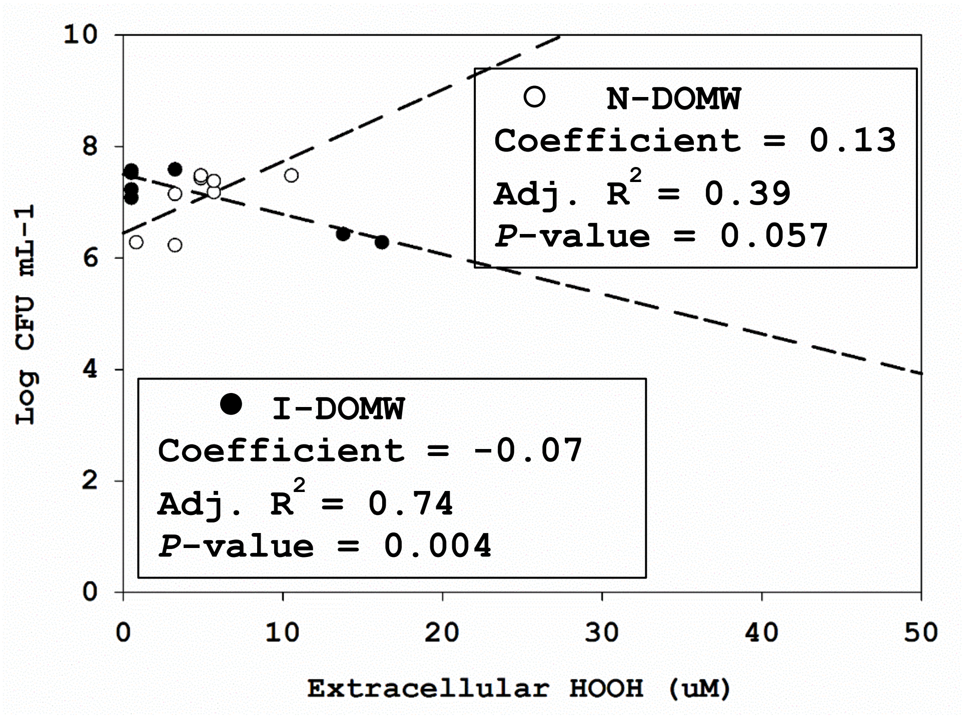


B

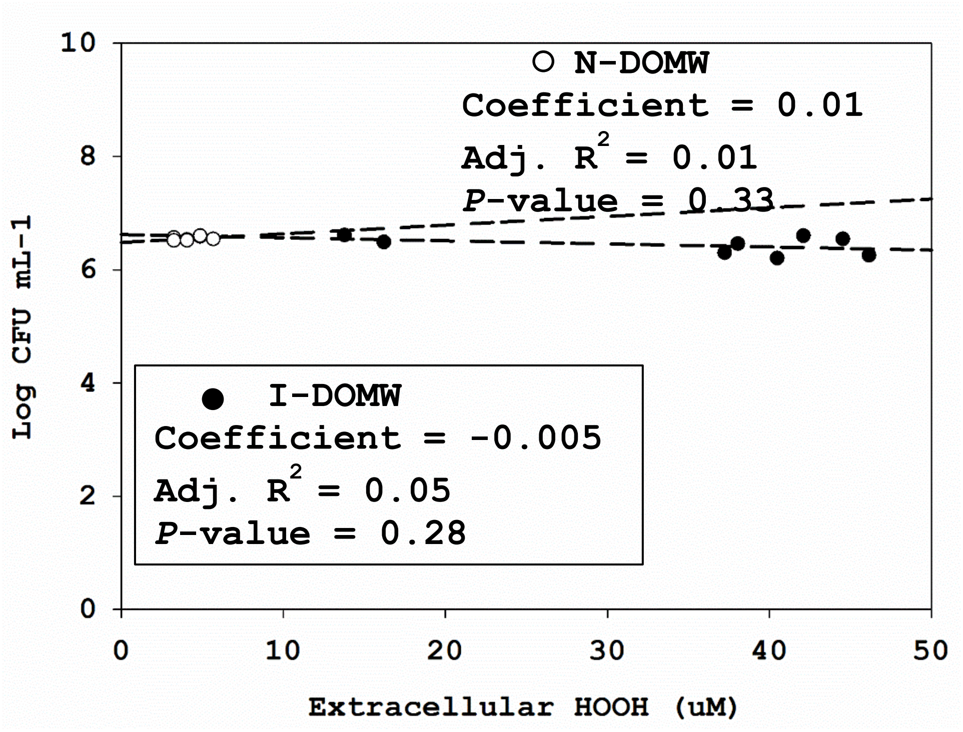

C

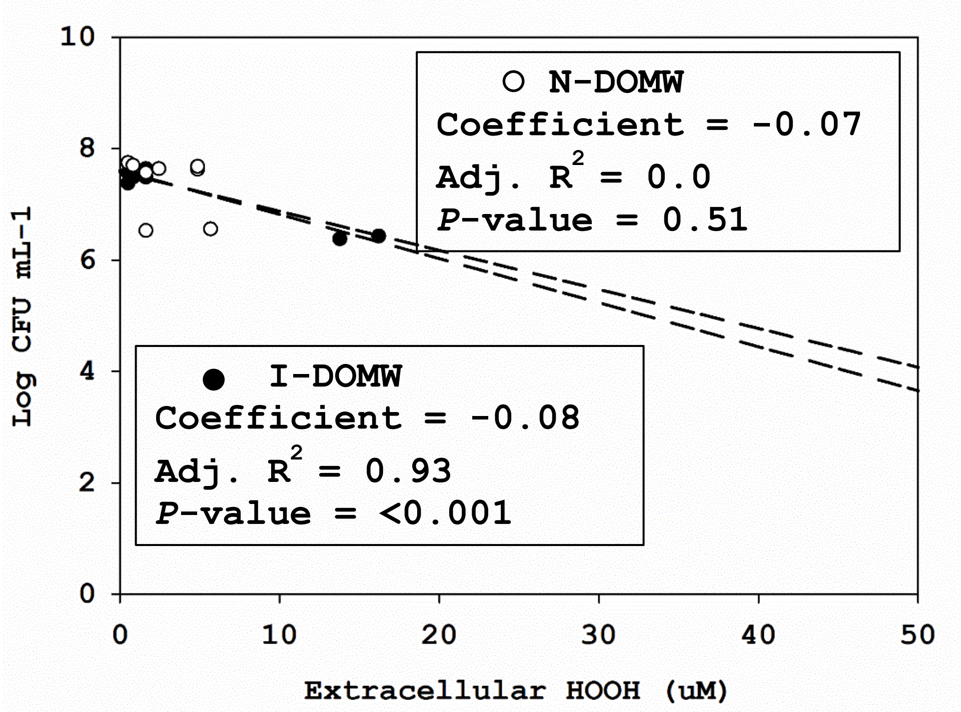


Figure S2. Linear regression of extracellular $H_2O_2$ concentration on bacteria concentration for (A) *E. coli* (B) *E. faecalis* and (C) *E. coli* O157: H7 at high inocula concentration (ca. $10^6$ CFU $ml^{-1}$).

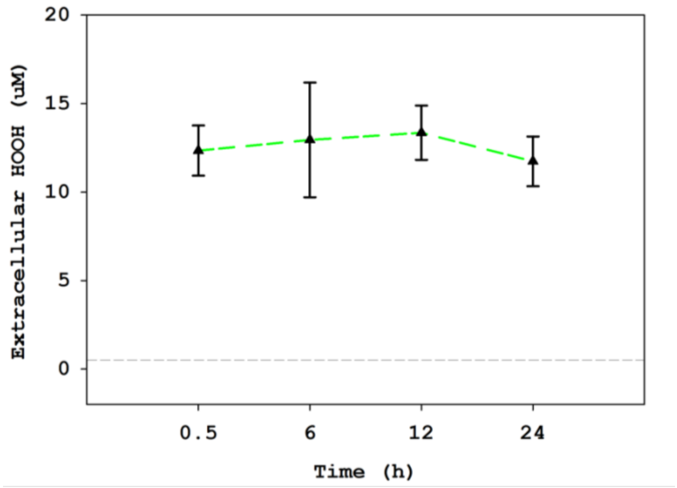


Figure S3: Extracellular $H_2O_2$ concentration in I-DOMW controls with no bacteria inoculation (n =3, 1 per experiment).

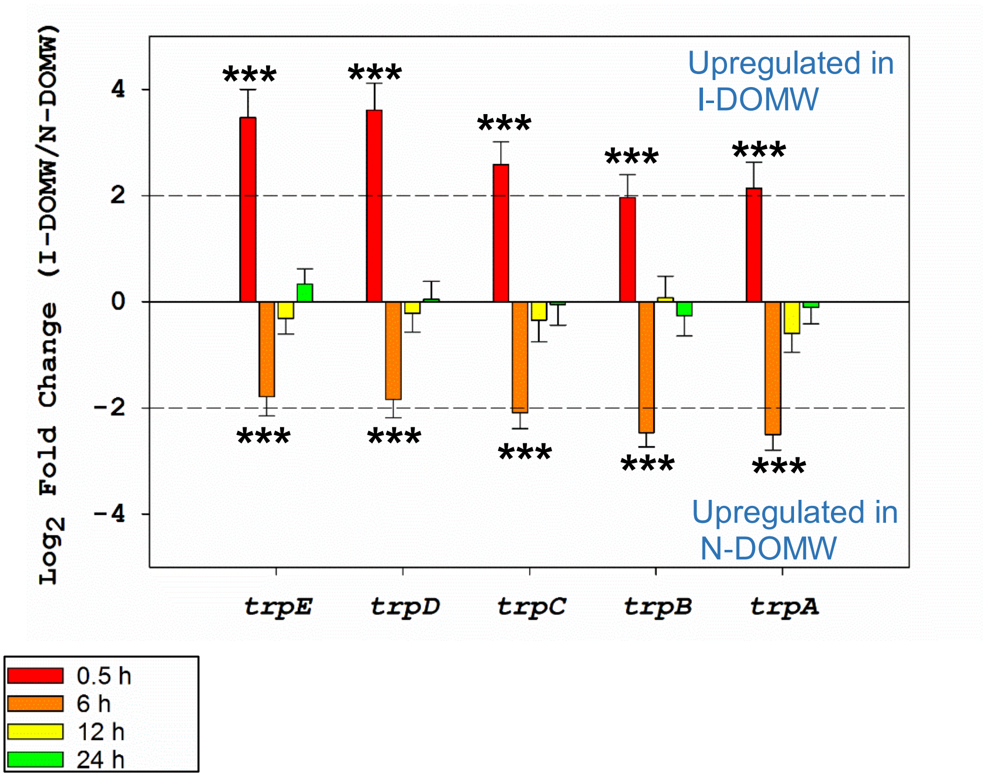


**Figure S4. Relative expression of indole-producing transcripts**. Fold change in expression of tryptophan operon transcripts for *E. coli* O157:H7 in I-DOMW relative to N-DOMW. Horizontal dash lines represent 2- fold change (***$p$.adj < 0.001).

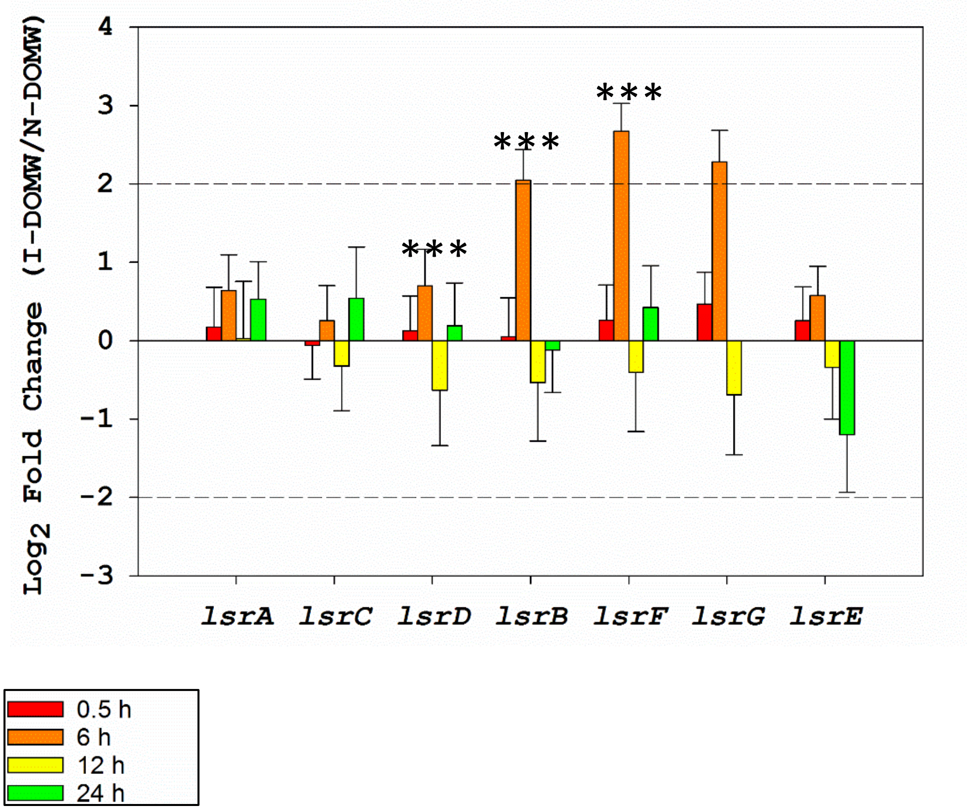


**Figure S5. Quorum sensing transcripts expression during dark incubation**. Fold change in expression of LuxS regulated *lsr* operon for *E. coli* in I-DOMW relative to N-DOM. Horizontal dash lines represent 2- fold change (****p*.adj < 0.001).

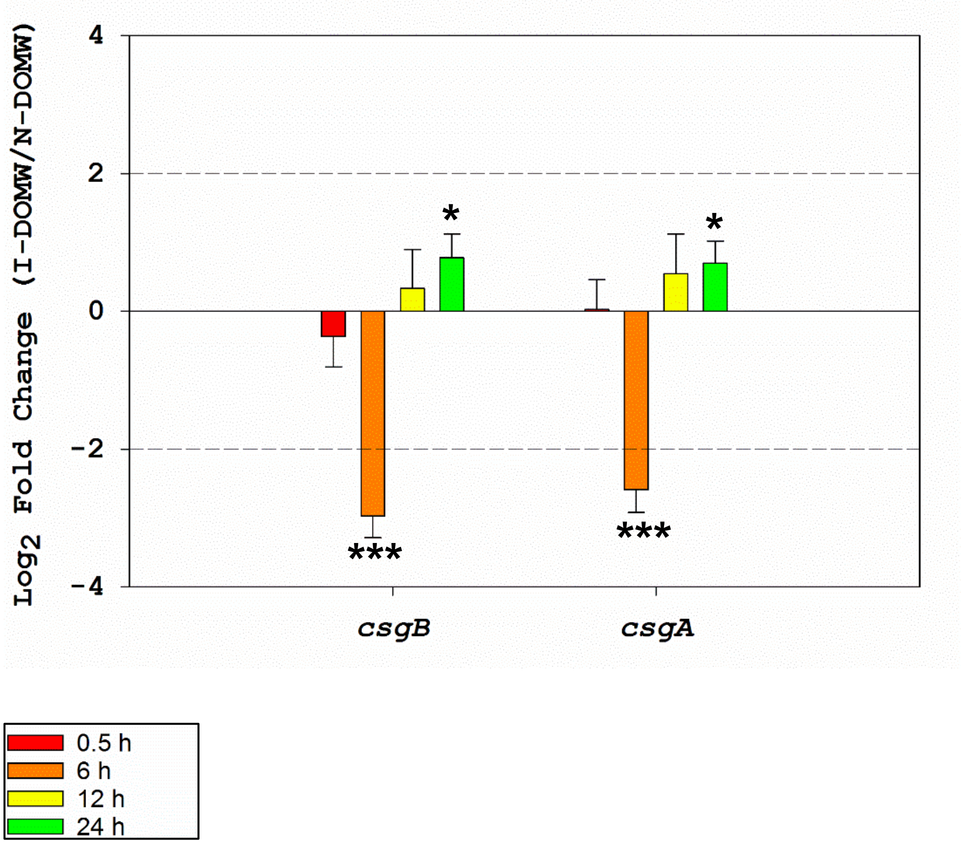


**Figure S6. Expression of biofilm forming transcripts.** Fold change in expression of *csgBA* operon (encoding curli) for *E. coli* in I-DOMW relative to N-DOMW. Horizontal dash lines represent 2- fold change (****p*.adj < 0.001).

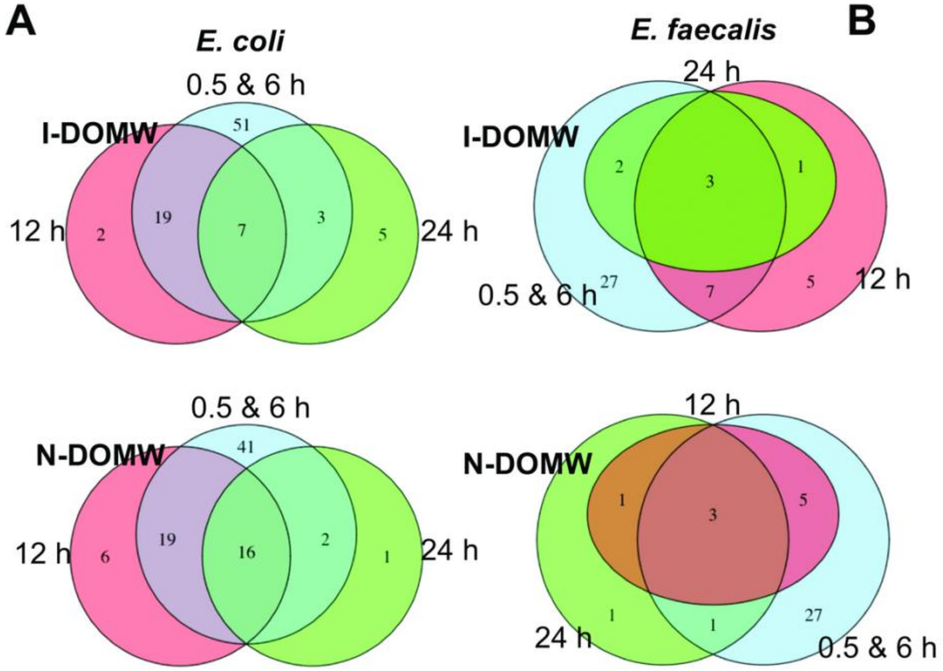


**Figure S7.** Venn diagram comparing number of Single Nucleotide Variants (SNV) shared between time points for (a) *E. coli* and (b) *E. faecalis* populations. SNVs were identified from RNA-seq data.

**Table S1**: Primers used for qRT-PCR

| Gene | Primers (5'-3') | Gene product name | Base pair (bp) | Amplification factor | Reference |
|---|---|---|---|---|---|
| *cysG* | F: ATGCGGTGAACTGTGGAATAAACG<br>R: TTGTCGGCGGTGGTGATGTC | Siroheme synthase | 105 | 1.98 | (Zhou et al., 2011) |
| *idnT* | F: CTGTTTAGCGAAGAGGAGATGC<br>R: ACAAACGGCGGCGATAGC | Gluconate permease | 90 | 2.00 | (Zhou et al., 2011) |
| *hcaT* | F: GGGCATTATGGGAGCAACTA<br>R: GGCAGAGTAAACCGCCTGTA | 3-phenyl propionic acid transporter | 154 | 1.93 | This study |
| *gapA* | F: GTCGCTGAAGCAACTGGTCT<br>R: AAGTTAGCGCCTTTAACGAACAT | Glyceraldehyde-3-phosphate dehydrogenase | 131 | 1.93 | (Michán et al., 1999) |
| *gyrB* | F: GCAAGCCACGCAGTTTCTC<br>R: GGAAGCCGACCTCTCTGATG | DNA gyrase subunit B | 254 | 2.05 | (Kyle et al., 2010) |
| *katG* | F: CTGCGTTTTGATCCTGAGTTC<br>R: GGCCCGATGTAGCGAGATT | Catalase peroxidase | 137 | 1.97 | (Michán et al., 1999) |
| *ahpF* | F: GCCCTGACCAAACTCTTTCC<br>R: GCAGATTCGCCATATTGACG | Alkyl hydroperoxide reductase subunit F | 204 | 1.76 | (Kyle et al., 2010) |
| *oxyS* | F: GAGCGGCACCTCTTTTAACCCTTG<br>R: CCTGGAGATCCGCAAAAGTTCACG | Oxidative stress regulator | 97 | 1.94 | (Kyle et al., 2010) |
| *oxyR* | F: CGCGATCAGGCAATGG<br>R: CAGCGCTGGCAGTAAAGTGAT | Hydrogen peroxide transcription regulator | 129 | 2.01 | (Michán et al., 1999) |
| *gyrB*[a] | F: ACACGGGTTCGTGAATTAGC<br>R: TTGGCTCTGGGAAAATAACG | DNA gyrase subunit B | 163 | 1.90 | This study |
| *rpoB*[a] | F: CCTGTACCTACCCACGGAGA<br>R: TGGACGTTTCACCAAAACAA | RNA polymerase subunit B | 150 | 1.90 | This study |
| *katA*[a] | F: TCTCATTCACCGGAAAGTCTTC<br>R: CAGCAGCATTGACCCATTTG | Catalase/Peroxidase | 121 | 2.07 | This study |
| *ahpC*[a] | F: CCAATTGGCTGGACAAACTT<br>R: ACGGTCAACTTGCTCGTTTC | Peroxiredoxin | 209 | 1.95 | This study |
| *npr*[a] | F: GGCCTAATCAAGACGGATGA<br>R: TCCTTGAACACCAGGGAAAG | NADH peroxidase | 195 | 1.83 | This study |
| *tpx*[a] | F: GGTTCCGACGAAACTTCTGA<br>R: GGTGTCGAAATGGAAATGCT | Thiol peroxidase | 161 | 1.70 | This study |
| *fur*[a] | F: CCAAACACTTTCACCACCAT<br>R: TTTGCTCTACTTCACCAAGCA | Ferric uptake regulation protein | 87 | 1.97 | (Riboldi et al., 2014) |
| *hypR*[a] | F: TCTCGACAAGCACAAGTTCC<br>R: ACCTAGCCCAGCTTCTACCA | Hydrogen peroxide regulator-arsR family protein | 126 | 2.03 | (Riboldi et al., 2014) |

Note. -

[a] Primers for *E. faecalis*

**Table S2**: Stability analysis of five commonly used reference genes in *E. coli* by geNorm (Vandesompele et al., 2002)

| Target | M value |
|---|---|
| *gapA* | 0.900 |
| *gyrB* | 0.900 |
| *cysG* | 1.542 |
| *hcaT* | 1.904 |
| *idnT* | 4.843 |

Note. - A lower 'M-value' correlates to higher gene expression stability.

**Table S3**: Multivariate analysis of variance between bacteria incubated in I-DOMW and N-DOMW at high inocula concentration

| **Bacteria** | **Treatment effect** | **F - value** | **Degrees of freedom (treatment, error)** | ***p*. value** |
|---|---|---|---|---|
| *E. coli* | $\mu_6 - \mu_{0.5}$ | 121 | 1, 2 | 0.0081 |
| | $\mu_{12} - \mu_6$ | 13 | 1, 2 | 0.069 |
| | $\mu_{24} - \mu_{12}$ | 0.4 | 1, 2 | 0.58 |
| | | | | |
| *E. faecalis* | $\mu_6 - \mu_{0.5}$ | 21.3 | 1, 2 | 0.043 |
| | $\mu_{12} - \mu_6$ | 3.6 | 1, 2 | 0.19 |
| | $\mu_{24} - \mu_{12}$ | 20.6 | 1, 2 | 0.045 |
| | | | | |
| *E. coli* O157:H7 | $\mu_6 - \mu_{0.5}$ | 1.1 | 1, 2 | 0.40 |
| | $\mu_{12} - \mu_6$ | 1.0 | 1, 2 | 0.42 |
| | $\mu_{24} - \mu_{12}$ | 0.4 | 1, 2 | 0.57 |
| | | | | |

Note. - High inoculum is ca. $10^6$ CFU $ml^{-1}$)

**Table S4**: Nutrient concentration before and after 12 h irradiation.

| | Before Irradiation (mean ± SD) | After Irradiation (mean ± SD) |
|---|---|---|
| $NH_4$ | 0.73±0.2 | 0.66±0.1 |
| $NO_3$ | 0.44±0.1 | 0.56±0.1** |
| DOC | 35.76±11.6 | 31.09±5.3 |
| $PO_4$ | 8.33±1.9 | 8.03±1.8 |

Note. - ** *p*. value < 0.001 denotes significant difference between concentration measured before and after sunlight irradiation of DOM-spiked water (Wilcoxon-signed rank test).
Standard deviation was derived from 4 technical replicates from 3 separate experiments.

**Table S5A.** Single Nucleotide Variants (SNV) identified in *E. coli* population.

| DOMW treatment[a] | Relative position | Allele in reference genome | Allele in DOMW population | QUAL[b] | Mutation | Gene name or Locus tag | Comments |
|---|---|---|---|---|---|---|---|
| I-DOMW | Intergenic | A | G | 42 | ND | ND | Upstream frr, downstream pyrH |
| I-DOMW | Intragenic | C | G | 47 | thr124thr | *yaiO* | outer membrane protein |
| I-DOMW | Intragenic | G | A | 36.01 | ala105ala | *mscK* | mechanosensitive channel protein, intermediate conductance, K+ regulated |
| I-DOMW | Intragenic | TC | T | 41.97 | ND | *ybhS* | putative ABC transporter permease |
| **I-DOMW** | **Intragenic** | **T** | **C** | **44** | **val410ala** | ***hyaB*** | **hydrogenase 1, large subunit** |
| I-DOMW | Intragenic | AGAAAATTAATGCAGCGCAGGATCTGAAAT | A | 51.97 | ND | *ydfC* | uncharacterized protein, Qin prophage |
| I-DOMW | Intergenic | A | AGGAAGGTG | 50.97 | ND | Intergenic | Upstream flhD, downstream IS1 transposase B |
| I-DOMW | Intergenic | C | CGA | 50.97 | ND | Intergenic | Upstream flhD, downstream IS1 transposase B |
| I-DOMW | Intergenic | A | G | 60 | ND | Intergenic | Upstream flhD, downstream IS1 transposase B |
| I-DOMW | Intergenic | T | C | 60 | ND | Intergenic | Upstream flhD, downstream IS1 transposase B |
| I-DOM | Intergenic | C | T | 43 | val260met | *yedQ* | putative membrane-anchored diguanylate |

| | | | | | | | |
|---|---|---|---|---|---|---|---|
| W | | | | | | | cyclase |
| I-DOMW | Intergenic | CA | C | 41.97 | ND | *mglC* | methyl-galactoside transport and galactose taxis; membrane component of ABC superfamily |
| I-DOMW | Intragenic | T | C | 102 | ND | *argZ* | tRNA-Arg |
| I-DOMW | Intragenic | A | T | 32.01 | val96glu | *obgE* | GTPase involved in cell partioning and DNA repair |
| I-DOMW | Intragenic | A | G | 51 | thr39ala | *yhjD* | inner membrane putative BrbK family alternate lipid exporter |
| I-DOMW | Intragenic | C | T | 44 | val33val | *rarD* | putative chloramphenical resistance permease |
| I-DOMW | Intragenic | A | C | 46 | asn485thr | *typA* | GTP-binding protein |
| N-DOMW | Intragenic | C | A | 34.01 | gly292cys | *paoC* | PaoABC aldehyde oxidoreductase, Moco-containing subunit |
| N-DOMW | Intragenic | CCG | C | 30.97 | ND | *entC* | isochorismate synthase 1 |
| N-DOMW | Intragenic | C | CA | 53.97 | ND | *dacC* | D-alanyl-D-alanine carboxypeptidase; penicillin-binding protein 6a |
| N-DOMW | Intragenic | CTG | C | 50.97 | ND | *mukB* | kinesin-like cell division protein involved in chromosome partitioning |
| N-DOMW | Intragenic | G | C | 60 | gln264his | *insQ* | IS609 transposase B |
| N-DOMW | Intragenic | G | T | 60 | arg267leu | *insQ* | IS609 transposase B |
| N-DOMW | Intragenic | GCC | G | 50.97 | ND | Intergenic | downstream of yncE and ansP |
| N-DOMW | Intragenic | G | A | 60 | ND | *ydfj* | pseudogene, MFS transporter family; interrupted by Qin prophage |
| N-DOMW | Intragenic | C | A | 60 | ND | *ydfj* | pseudogene, MFS transporter family; interrupted by Qin prophage |
| N-DOM | Intergenic | T | A | 47 | ND | Intergenic | Upstream ydiE, downstream aroH |

| | | | | | | | |
|---|---|---|---|---|---|---|---|
| W | | | | | | | |
| N-DOMW | Intragenic | G | A | 56 | arg105arg | *nuoC* | NADH:ubiquinone oxidoreductase, fused CD subunit |
| N-DOMW | Intragenic | CCAG | C | 50.97 | ND | *der* | putative GTP-binding factor |
| N-DOMW | Intragenic | C | A | 37.01 | gly342cys | *mltA* | membrane-bound lytic murein transglycosylase A |
| N-DOMW | Intragenic | GA | G | 31.97 | ND | *nanA* | N-acetylneuraminate lyase (aldolase |
| N-DOMW | Intragenic | CGCT | C | 50.97 | ND | *dppD* | putative ATP-binding component of dipeptide transport system |
| N-DOMW | Intragenic | C | A | 43 | val550leu | *ilvB* | acetolactate synthase I,valine-sensitive, large subunit |
| N-DOMW | Intragenic | CGG | C | 30.97 | ND | *zraS* | sensory histidine kinase in two-component regulatory system with ZraR |
| N-DOMW | Intragenic | CT | C | 31.97 | ND | *nrfD* | formate-dependent nitrite reductase, membrane subunit |
| N-DOMW | Intragenic | AC | A | 41.97 | ND | *yjgM* | GNAT family putative N-acetyltransferase |
| N-DOMW | Intragenic | G | A | 47 | his144his | *fecI* | RNA polymerase sigma-19 factor, fec operon-specific; ECF sigma factor |

**Table S5B.** Single Nucleotide Variants (SNV) identified in *E. faecalis* population.

| **DOMW treatment[a]** | **Relative position** | **Allele in reference genome** | **Allele in DOMW population** | **QUAL[b]** | **Mutation** | **Gene name or Locus tag** | **Comments** |
|---|---|---|---|---|---|---|---|
| I-DOMW | Intragenic | A | G | 75 | met865Ile | DR75_184 | PD-(D/E)XK nuclease superfamily protein |
| I-DOMW | Intragenic | G | T | 46 | leu976phe | DR75_393 | putative CoA-substrate-specific enzyme activase domain protein |
| I-DOMW | Intragenic | C | A | 38.01 | asp220ile | *purF* | amidophosphoribosyltransferase |
| I-DOMW | Intragenic | C | A | 52 | ser371ser | DR75_1102 | aminotransferase class I and II family protein |
| I-DOMW | Intragenic | C | T | 52 | gln330stop | DR75_977 | heme ABC exporter, ATP-binding protein CcmA |

| | | | | | | | |
|---|---|---|---|---|---|---|---|
| I-DOMW | Intergenic | C | CA | 70.97 | ND | DR75_1948 | Upstream of rpoB and downstream of pemK-like family protein G26 |
| I-DOMW | Intergenic | G | T | 30.01 | ND | ND | downstream inosine-5'-monophosphate dehydrogenase and serS: serine--tRNA ligase |
| I-DOMW | Intragenic | A | G | 52 | trp19arg | DR75_2040 | oxaloacetate decarboxylase, gamma chain family protein |
| I-DOMW | Intragenic | A | G | 33.01 | ND | DR75_2299 | tRNA-Arg |
| N-DOMW | Intragenic | T | A | 49 | Asn81tyr | *murB* | UDP-N-acetylenolpyruvoylglucosamine reductase |
| N-DOMW | Intergenic | CTT | C | 30.97 | ND | ND | Upstream of DR75_2212 (hypotethical) |
| **N-DOMW** | **Intragenic** | **G** | **A** | **407.5** | **glu269glu** | **DR75_2888** | **putative LPXTG-domain-containing protein cell wall anchor domain** |

Note. - SNVs were identified from RNA-seq data collected after 0.5, 6, 12 and 24 h of dark incubation in DOM-spiked water.
**Boldness** denotes mutations discussed in the manuscript.
[a]I-DOMW - Irradiated DOM-spiked Water, N-DOMW - Non-irradiated DOM-spiked Water
[b]QUAL - phred-scaled quality score (high QUAL scores indicate high confidence in SNV identified).

**Table S6.** RNA-seq coverage for *E. coli* and *E. faecalis* populations.

| RNA-seq sample_ID | Chromosome (X) | pTEF3_66kb (X) | pAD1_41kb (X) |
|---|---|---|---|
| EC3000 IDOMW 0.5 h | 231 | | |
| EC3000 IDOMW 6 h | 67 | | |
| EC3000 IDOMW 6 h | 70 | | |
| EC3000 IDOMW 12 h | 60 | | |
| EC3000 IDOMW 24 h | 33 | | |
| EC3000 NDOMW 0.5 h | 80 | | |
| EC3000 NDOMW 0.5 h | 57 | | |
| EC3000 NDOMW 6 h | 90 | | |
| EC3000 NDOMW 6 h | 127 | | |
| EC3000 NDOMW 12 h | 87 | | |
| EC3000 NDOMW 12 h | 84 | | |
| EC3000 NDOMW 24 h | 57 | | |
| EC3000 NDOMW 24 h | 96 | | |
| EF29212 IDOMW 0.5 h | 55 | 6 | 3 |
| EF29212 IDOMW 6 h | 213 | 10 | 6 |
| EF29212 IDOMW 6 h | 170 | 24 | 13 |
| EF29212 IDOMW 12 h | 166 | 7 | 5 |
| EF29212 IDOMW 12 h | 153 | 4 | 4 |
| EF29212 IDOMW 24 h | 245 | 8 | 8 |
| EF29212 NDOMW 0.5 h | 172 | 13 | 8 |
| EF29212 NDOMW 6 h | 179 | 23 | 15 |
| EF29212 NDOMW 12 h | 108 | 3 | 2 |
| EF29212 NDOMW 24 h | 95 | 3 | 3 |

Note. - Coverage was determined by mapping fastq files to corresponding reference genomes using geneious v. 10 with default parameters.
EC3000 – *E. coli* C3000, EF29212 – *E. faecalis* ATCC strain 29212
I-DOMW - Irradiated DOM-spiked Water, N-DOMW - Non-irradiated DOM-spiked Water

## Supplementary Files

Supplementary File 1: Excel workbook with metadata on all growth experiments conducted.

Supplementary File 2: Excel workbook with read numbers.

Supplementary File 3: Excel workbook with differential gene expression as determined by DESeq2.

Supplementary File 4: Microsoft word document with bioinformatics scripts.

## REFERENCES


Chen, C.-Y., and Jafvert, C.T. (2010). Photoreactivity of carboxylated single-walled carbon nanotubes in sunlight: reactive oxygen species production in water. *Environmental science & technology* 44(17)**,** 6674-6679.

Crumpton, W.G., Thomas, I.M., and Mitchell Paul, D. (1992). Nitrate and organic N analyses with second-derivative spectroscopy. *Limnol. Oceanogr* 37(4)**,** 907-909.

Haag, W.R., and Hoigne, J. (1986). Singlet oxygen in surface waters. 3. Photochemical formation and steady-state concentrations in various types of waters. *Environmental science & technology* 20(4)**,** 341-348.

Haag, W.R., and Hoigné, J. (1985). Photo-sensitized oxidation in natural water via. OH radicals. *Chemosphere* 14(11-12)**,** 1659-1671.

Kosaka, K., Yamada, H., Matsui, S., Echigo, S., and Shishida, K. (1998). Comparison among the methods for hydrogen peroxide measurements to evaluate advanced oxidation processes: application of a spectrophotometric method using copper (II) ion and 2, 9-dimethyl-1, 10-phenanthroline. *Environmental science & technology* 32(23)**,** 3821-3824.

Kyle, J.L., Parker, C.T., Goudeau, D., and Brandl, M.T. (2010). Transcriptome analysis of Escherichia coli O157: H7 exposed to lysates of lettuce leaves. *Applied and environmental microbiology* 76(5)**,** 1375-1387.

Michán, C., Manchado, M., Dorado, G., and Pueyo, C. (1999). In vivo transcription of the Escherichia coli oxyRregulon as a function of growth phase and in response to oxidative stress. *Journal of bacteriology* 181(9)**,** 2759-2764.

Riboldi, G.P., Bierhals, C.G., Mattos, E.P.d., Frazzon, A.P.G., and Frazzon, J. (2014). Oxidative stress enhances the expression of sulfur assimilation genes: preliminary insights on the Enterococcus faecalis iron-sulfur cluster machinery regulation. *Memórias do Instituto Oswaldo Cruz* 109(4)**,** 408-413.

Vandesompele, J., De Preter, K., Pattyn, F., Poppe, B., Van Roy, N., De Paepe, A., et al. (2002). Accurate normalization of real-time quantitative RT-PCR data by geometric averaging of multiple internal control genes. *Genome biology* 3(7)**,** research0034.

Zhou, K., Zhou, L., Lim, Q.E., Zou, R., Stephanopoulos, G., and Too, H.-P. (2011). Novel reference genes for quantifying transcriptional responses of Escherichia coli to protein overexpression by quantitative PCR. *BMC molecular biology* 12(1)**,** 18.
