# Supplementary material for: Transcriptome Changes of Escherichia coli, Enterococcus faecalis, and Escherichia coli O157:H7 Laboratory Strains in Response to Photo-Degraded DOM
Source: Front Microbiol. 2018 May 8;9:882. doi: 10.3389/fmicb.2018.00882 (PMC5953345; doi:10.3389/fmicb.2018.00882)
Supplement: Supplementary file 5 [file Data_Sheet_5.DOCX]

**Supplementary File 4**: Microsoft word document with bioinformatics scripts.

**##### Unix command line was used on the Georgia Advanced Computing Resource Center (GACRC) cluster #####**

**####Map reads to reference genome -R is to denote read groups and sample id, -t is for thread####**

#!/bin/bash

cd /escratch4/ade1986/ade1986_Mar_31/RNAseq_Regrowth/

/usr/local/bwa/latest/bwa mem -t 8 -R "@RG\tID:EO157\tSM:Time_6h_I-DOMW_1\tPL:Illumina_Nextseq\tLB:PE" NC_002655.fna ECO157_B6914_I-DOMW_6h_S5_R1_001.fastq ECO157_B6914_I-DOMW_6h_S5_R2_001.fastq > ECO157_B6914_I-DOMW_6h_1.sam

/usr/local/bwa/latest/bwa mem -t 8 -R "@RG\tID:EO157\tSM:Time_6h_I-DOMW_2\tPL:Illumina_Nextseq\tLB:PE" NC_002655.fna ECO157_B6914_I-DOMW_6h_S6_R1_001.fastq ECO157_B6914_I-DOMW_6h_S6_R2_001.fastq > ECO157_B6914_I-DOMW_6h_2.sam

/usr/local/bwa/latest/bwa mem -t 8 -R "@RG\tID:EO157\tSM:Time_6h_N-DOMW_1\tPL:Illumina_Nextseq\tLB:PE" NC_002655.fna ECO157_B6914_N-DOMW_6h_S12_R1_001.fastq ECO157_B6914_N-DOMW_6h_S12_R2_001.fastq > ECO157_B6914_N-DOMW_6h_1.sam

/usr/local/bwa/latest/bwa mem -t 8 -R "@RG\tID:EO157\tSM:Time_6h_N-DOMW_2\tPL:Illumina_Nextseq\tLB:PE" NC_002655.fna ECO157_B6914_N-DOMW_6h_S11_R1_001.fastq ECO157_B6914_N-DOMW_6h_11_R2_001.fastq > ECO157_B6914_N-DOMW_6h_2.sam

**####Convert SAM to BAM####**

samtools view -bS ECO157_B6914_I-DOMW_6h_1.sam > ECO157_B6914_I-DOMW_6h_1.bam

samtools view -bS ECO157_B6914_I-DOMW_6h_2.sam > ECO157_B6914_I-DOMW_6h_2.bam

samtools view -bS ECO157_B6914_N-DOMW_6h_1.sam > ECO157_B6914_N-DOMW_6h_1.bam

samtools view -bS ECO157_B6914_N-DOMW_6h_2.sam > ECO157_B6914_N-DOMW_6h_2.bam

**####Sort BAM files by coordinate####**

samtools sort ECO157_B6914_I-DOMW_6h_1.bam ECO157_B6914_I-DOMW_6h_1_sorted

samtools sort ECO157_B6914_I-DOMW_6h_2.bam ECO157_B6914_I-DOMW_6h_2_sorted

samtools sort ECO157_B6914_N-DOMW_6h_1.bam ECO157_B6914_N-DOMW_6h_1_sorted

samtools sort ECO157_B6914_N-DOMW_6h_2.bam ECO157_B6914_N-DOMW_6h_2_sorted

**####Remove PCR artifacts/duplicates####**

/usr/local/samtools/0.1.9/samtools rmdup ECO157_B6914_I-DOMW_6h_1_sorted.bam ECO157_B6914_I-DOMW_6h_1_rmdup.bam

/usr/local/samtools/0.1.9/samtools rmdup ECO157_B6914_I-DOMW_6h_2_sorted.bam ECO157_B6914_I-DOMW_6h_2_rmdup.bam

/usr/local/samtools/0.1.9/samtools rmdup ECO157_B6914_N-DOMW_6h_1_sorted.bam ECO157_B6914_N-DOMW_6h_1_rmdup.bam

/usr/local/samtools/0.1.9/samtools rmdup ECO157_B6914_N-DOMW_6h_2_sorted.bam ECO157_B6914_N-DOMW_6h_2_rmdup.bam

**####Index BAM files, required for bedtools####**

samtools index -b ECO157_B6914_I-DOMW_6h_1_rmdup.bam > ECO157_B6914_I-DOMW_6h_1

samtools index -b ECO157_B6914_I-DOMW_6h_2_rmdup.bam > ECO157_B6914_I-DOMW_6h_2

samtools index -b ECO157_B6914_N-DOMW_6h_1_rmdup.bam > ECO157_B6914_N-DOMW_6h_1

samtools index -b ECO157_B6914_N-DOMW_6h_2_rmdup.bam > ECO157_B6914_N-DOMW_6h_2

**####Count alignment using BEDtools####**

/usr/local/bedtools/latest/bin/multiBamCov -bams ECO157_B6914_I-DOMW_6h_1_rmdup.bam ECO157_B6914_I-DOMW_6h_2_rmdup.bam ECO157_B6914_N-DOMW_6h_1_rmdup.bam ECO157_B6914_N-DOMW_6h_2_rmdup.bam -bed NC_002655.gff > ECO157_B6914_6h_gene_counts.gff

**##### Differential gene expression was analyzed in R #####**

**###Load Bioconductor package/DESeq2###**

library(DESeq2)

counts <- read.csv("ECO157_B6914_6h_gene_counts.csv",header=FALSE,

row.names=1)

colnames(counts) <- c("I-DOMW1","I-DOMW2","N-DOMW1", "N-DOMW2")

conditions <- c(rep("I-DOMW",2), rep("N-DOMW", 2))

ECO157_6h.ds<- DESeqDataSetFromMatrix(countData = counts,colData = as.data.frame(conditions), design = ~ conditions)

colData(ECO157_6h.ds)$conditions <- factor(colData(ECO157_6h.ds)$conditions, levels=c("N-DOMW", "I-DOMW")) **##N-DOMW is reference##**

ECO157_6h.ds <- ECO157_6h.ds[ rowSums(counts(ECO157_6h.ds)) > 1, ] ##(Removes reads with 0 or 1 count)##

ECO157_6h.ds <- DESeq(ECO157_6h.ds)

res <- results(ECO157_6h.ds)

summary (res)

**###Exporting result as CSV###**

write.csv(as.data.frame(res),file="ECO157_B6914_6h_DESeq2.csv")
